# Neuropixels reveal laminar microcircuit organization in monkey V1 in vivo

**DOI:** 10.1073/pnas.2521556123

**Published:** 2026-02-18

**Authors:** Nicole Carr, Shude Zhu, Xiaomo Chen, Eric Kenji Lee, Alec Perliss, Tirin Moore, Chandramouli Chandrasekaran

**Affiliations:** ^a^Department of Biomedical Engineering, Boston University, Boston, MA 02115; ^b^Department of Neurobiology, Stanford University School of Medicine, Stanford, CA 94305; ^c^HHMI, Stanford, CA 94305-5323; ^d^Department of Neurobiology, Center for Neuroscience, University of California, Davis, CA 95618; ^e^Department of Psychological and Brain Sciences, Boston University, Boston, MA 02115; ^f^Center for Systems Neuroscience, Boston University, Boston, MA 02115; ^g^Department of Anatomy and Neurobiology, Boston University, Boston, MA 02118

**Keywords:** high-resolution electrophysiology, visual cortex, primate, neocortex, neural circuits

## Abstract

The relationship between different cell populations in monkey primary visual cortex and their role in visual function is not fully resolved. We combined high-density Neuropixels recordings across layers of macaque V1, and a state-of-the-art nonlinear dimensionality reduction approach on waveform shape to delineate nine putative cell classes: 4 narrow-spiking (NS), 4 broad-spiking (BS), and 1 triphasic (TP). Then, we performed targeted analyses of laminar organization, spike amplitude, multichannel spatial features, functional properties, and network connectivity of these cell classes. These analyses have uncovered four fundamental aspects of V1 laminar microcircuitry never fully demonstrated before in vivo. First, NS neurons were most concentrated in layer 4 and outnumbered parvalbumin positive neurons, consistent with findings on potassium channel expression in excitatory neurons in V1. Second, a large-amplitude NS cell class in layer 4b was strongly direction selective, with multichannel waveforms suggestive of a stellate morphology, a likely functional correlate of anatomical descriptions of neurons projecting from V1 to MT. Third, another NS cell class in layer 4b showed robust bursting activity and strong orientation selectivity. Finally, cross-correlation analysis revealed distinct feedforward interactions between cell classes in layer 4 and layer 5/6 and those in layer 2/3. These results demonstrate how high-resolution electrophysiology can reveal links between laminar organization and in vivo function of neurons. Our findings offer key insights for biologically realistic microcircuit models of primate V1 and may generalize to other regions.

## Significance

This study employs an innovative approach that combines high-density electrophysiology and machine learning to link in vivo structure with visual function in the monkey primary visual cortex. It is a demonstration of how high-resolution electrophysiology can reveal relationships between the structural organization and in vivo function of neurons and also provides key insights for biologically realistic microcircuit models of the primate visual cortex.

The primary visual cortex (V1) of the monkey is the first cortical area involved in vision and has been the subject of intense anatomical, in vitro, and in vivo research over the past 50 y (1–9). Single-cell RNA-seq analyses have recently identified 13 transcriptomically separable excitatory cell classes, and 5 inhibitory classes in macaque V1 (10). These different neuron types are located in different laminae and sublaminae (1), possess distinct morphology (11, 12), express a diversity of ion channels (13, 14), and interlaminar connection patterns (6). Other studies have provided insight into the morphological properties of neurons that connect to other visual areas such as MT and V2 (15–19). In parallel, in vitro studies in the cat and monkey have delineated the firing properties of neurons in various layers of the cortex (11, 20) and identified bursting neural populations (21, 22). Finally, neurophysiological experiments have identified tuning to diverse visual features (e.g., orientation, color, and direction) in V1 neurons (23–27), and even described functional cell types in vivo (22, 28).

However, technical limitations meant that these studies could not fully characterize how specific cell types within V1 layers contribute to visual function. Single-electrode recordings typically record from one neuron at a time, often targeting the largest neurons. In addition, these recordings focused on the somatic action potential, precluding insight into how the action potentials change across compartments of a neuron. Recent modeling and high-density electrophysiology studies suggest that recording from a neuron on multiple linear channels can capture the spatiotemporal dynamics of action potential propagation (29). This includes differences in propagation velocity along the probe-which may reflect the morphology, that is the dendritic and axonal architecture of the cell, and improve cell type identification (30–33). Moreover, datasets with small neuron yield pooled across multiple animals could introduce variability that can obscure layer- and cell-type-specific patterns. Additionally, analysis approaches that rely solely on somatic waveform width to separate putative inhibitory (narrow-spiking) from excitatory (broad-spiking) neurons can be misleading, as some excitatory neurons are narrow-spiking and some inhibitory neurons are broad-spiking (13, 34–36). These limitations meant that past studies could not fully characterize the diversity of neuronal populations or assess functional relationships between simultaneously active neurons across layers of V1.

Our goal was to improve our understanding of macaque V1’s laminar microcircuitry by capitalizing on two recent advances: 1) neuropixels technology and 2) novel machine learning approaches for identifying cell types from waveforms.

Neuropixels probes consist of densely spaced recording sites (20 μm spacing) that enable simultaneous recording of populations of neurons across cortical layers, which are well defined in V1. This higher spatial sampling enables detection of small waveforms, particularly those of axons or small neurons (e.g., stellate cells in L4c). Individual neurons are also detected across multiple adjacent channels, capturing the spatiotemporal propagation of action potentials above and below the soma. Neuropixels provide improved spatiotemporal sampling, and allow some inferences of cell-type specific morphological features, thereby informing a more complete understanding of the microcircuit in V1.

We applied our WaveMAP machine learning approach to normalized extracellular waveforms to delineate candidate cell types (34, 37). We then organized these candidate cell types by laminar position within the cortex, revealing functional microcircuit architecture in V1 that was previously inaccessible without the combination of high-resolution electrophysiology and machine learning-based cell-type classification.

Understanding whether cell populations across layers have distinct functional and physiological properties—and whether those populations can be identified by their electrophysiological signatures—is critical for understanding visual computation across V1’s microcircuit. For example, layer 4 and layer 6, are primary recipients of thalamic input and early cortical stages where orientation and direction selectivity emerge. Consistent with our goal, we use various analyses to demonstrate that amplitude, morphology, laminar organization, bursting, and waveform shape are related to functional properties such as orientation and direction selectivity (38). Our results suggest that high-density recordings and machine learning-based cell type classification will enable further tests of hypotheses about how cell populations in V1 mediate visual function (39–43). Linking neuronal diversity to functional specialization will advance biologically realistic models of V1 and help explain how cell type diversity contributes to functional microcircuits across the neocortex.

## Results

**Fig. 1.**
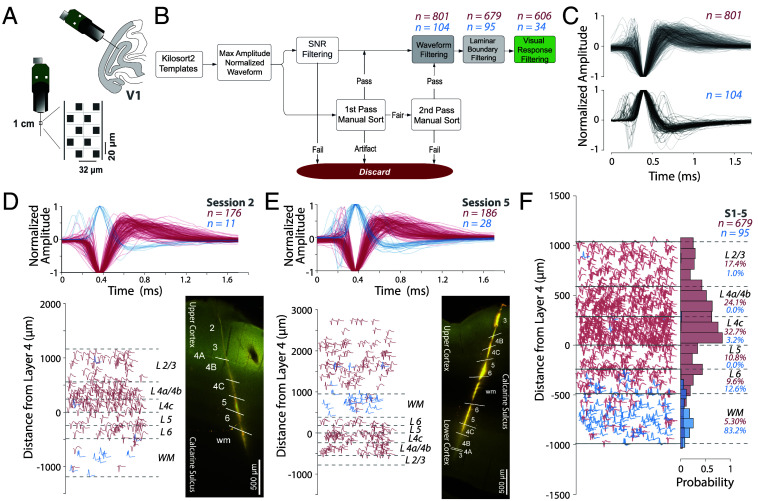
Neuropixels recordings reveal rich neural populations across layers of V1. (*A*) *Upper*, The angle of probe penetrations made into the lateral surface and underlying calcarine sulcus of V1 (see *SI Appendix*, Fig. S1 *A*–*D* and *Electrophysiology Recordings* for precise recording locations). *Lower Left*, Neuropixels 1.0 probe base and shank. The *Inset* shows layout of electrode contacts for a section of the recording shank. (*B*) Semiautomatic quality control process applied to single unit templates extracted from Kilosort2 analysis to identify single neurons. This process includes the following steps: 1) Identify the maximum amplitude channel from the waveform template and then normalize between −1 and 1 for each neuron. 2) Manually curate each normalized waveform. We used two independent observers for manual curation of the waveform templates with an interrater reliability of 0.72. Each observer performed two passes of curation. 3) Filter out artifacts through SNR thresholding. 4) Combine waveforms that passed manual curation and SNR threshold into dataset. (*pink*: negative spiking units, *blue*: positive spiking units). (*C*) *Upper*, 801 negative spiking extracellular waveforms after quality control. *Lower*, 104 positive spiking extracellular waveforms. (*D*) Example Session 2, Monkey 1 (M1), *Upper*, 187 waveforms across all layers of V1 (*pink*: negative spiking units, *blue*: positive spiking units). *Lower Left*, Laminar distribution with marked layer boundaries, determined by current source density and histology. *Lower Right*, Histology image of probe track. (*E*) Example Session 5, Monkey 2 (M2), *Upper*, 214 waveforms across all layers of V1 (*pink*: negative spiking units, *blue*: positive spiking units). *Lower Left*, Laminar distribution with marked layer boundaries, determined by current source density and histology. The layers here are flipped because the session 5 recording was taken from the deeper cortex. *Lower Right*, Histology image of probe track through calcarine sulcus into the cortex below. (*F*) Pooled waveforms within average laminar boundaries from M1 and M2, sessions 1 to 5, *Right*, Distribution of 774 waveforms across all layers of the cortex with scaled depths (*pink*: 679 negative spiking units, *blue*: 95 positive spiking units).

Our goal was to use Neuropixels to further understand laminar microcircuits in monkey V1. We reanalyzed a recent dataset (28, 44), with high-resolution Neuropixels recordings from the primary visual cortex (V1) of two isoflurane anesthetized macaque monkeys (5 sessions, M1 [sessions 1 to 3] and M2 [sessions 4 to 5], Fig. 1*A* and *SI Appendix*, *Surgical Details*). The Neuropixels probes were coated with DiI to facilitate histological analysis and delineation of layer boundaries. For monkey M1, recordings were nearly perpendicular to the lateral opercular V1 surface and largely restricted to superficial cortex (*SI Appendix*, Fig. S1 *A* and *B*). For monkey M2, the probe penetrated through the surface opercular V1 and white matter, and reached the deep calcarine V1 cortex below (*SI Appendix*, Fig. S1 *C* and *D* and *Electrophysiology Recordings*). We used current source density (CSD) to determine the L4c and L5 boundary, and then used histological measurements to assign neurons to layers (*SI Appendix*, *Laminar Boundary Assignment*). We combined L2/L3, L4a/L4b, L4c*α*/L4c*β*, and L5/L6 to ensure that we had sufficient power for our analyses (*SI Appendix*, Table S1) as splitting further would have meant we had no units in some layers. A recent study (45) suggested that CSD was variable and only moderately useful for laminar assignment and instead recommended action potential based measures to identify layers. We mitigated this issue by combining histology with CSD to accurately delineate the laminar boundaries (*SI Appendix*, Fig. S1 *A*–*D*), avoiding the circular inference of using waveforms to both identify layers and assign cell types to those layers.

We recorded broadband extracellular activity in V1 while stimulating with drifting Gabor gratings in 36 directions, 4 different spatial frequencies, and either monocular or monocular and binocular conditions (*SI Appendix*, *Visual Stimulation*). We only used the optimal eye conditions for analysis.

### Rigorous Spike Sorting to Identify Single Units.

We used Kilosort2 to spike sort the broadband extracellular data and extract template waveforms representing the extracellular action potential trace from each unit (46). After spike sorting, we obtained a total of 2,529 template waveforms. We then used a rigorous, semiautomated, and conservative waveform curation process (Fig. 1*B* and *SI Appendix*, *Spike Sorting and Data Curation*) and identified 905 single neurons (*SI Appendix*, Fig. S1*E*). We separated neurons into “positive spiking,” where the (hyperpolarization) peak of the waveform came before the trough (depolarization) and “negative spiking,” where the trough came before the peak. The positive spiking neurons were aligned to the peak, and the negative spiking neurons to the trough. In the case of triphasic waveforms, where two major peaks were detected on either side of the trough, the waveforms were aligned to the trough and second peak, and were classified as “negative spiking” waveforms.

We identified 801 negative spiking neurons, and 104 positive spiking neurons from all recording sessions (Fig. 1*C*, *Upper* and *Lower* panels respectively, *SI Appendix*, Fig. S1*E*). Since we were comparing the waveform to functional properties, we only selected neurons that were responsive to visual stimuli. Therefore, for monkey M2, we excluded the neurons from surface V1, because the visual stimulation was performed based on the RF locations of the calcarine sulcus V1 (*SI Appendix*, Fig. S1 *C*, *D*, and *G*). 606 negative spiking units were both responsive to visual stimuli and within the layer boundaries from L2/3 to white matter (*SI Appendix*, Fig. S1 *F* and *G*). These negative spiking units were used as the primary dataset for furthering our understanding of the functional microcircuit in V1.

### Waveforms in White Matter Are Largely Positive-Spiking.

Overall, negative spiking neurons were more common in gray matter (layers 1 to 6, pink, $\chi^{2}$ (1, 679) = 542.63, P<0.0001, Fig. 1*F* and *SI Appendix*, Fig. S1 *F* and *G*), whereas the positive spiking neurons were more common in white matter (blue, $\chi^{2}$ (1, 95) = 41.78, P<0.0001, Fig. 1*F*), consistent with a recent study of the distribution of positive spiking waveforms in V1 (45). In monkey M1, the recordings were largely restricted to the opercular surface of V1, and the positive waveforms were largely found in the white matter (blue, Fig. 1*D*). Even in monkey M2, where recordings covered the opercular cortex, white matter, and cortex below the calcarine sulcus, positive waveforms were more common in the white matter (Fig. 1*E*). However, very few positive spiking neurons were visually responsive and so were excluded from the functional analyses (*SI Appendix*, *Supplementary Discussion* and Fig. S6 *A*–*E*).

Fig. 1*F* summarizes this pattern from all five sessions in a normalized space with layer boundaries. To preserve the depth of the neurons within their original layer boundaries (*SI Appendix*, *Scaling Laminar Depths*), we first calculated the average layer boundaries of the five sessions. Then, we scaled the depths of each neuron to fit within the average layer boundaries. Finally, for each neuron in each session, we identified the layer in which the neuron was present. In our dataset, due to the depth of the probe penetration, neurons in L2/3 in M2 were undersampled (Fig. 1*F* and *SI Appendix*, Fig. S1 *F* and *G*). Nevertheless, in both monkeys, we still observed lower cell density in L5/6 compared to L4c, consistent with previous studies (14, 45). Orientation tuning curves for all the visually responsive neurons (*SI Appendix*, Fig. S1*H*) were similar suggesting our recordings were largely perpendicular to the cortical surface (28, 44).

We examined the overall visual response properties (*SI Appendix*, *Receptive Field Properties*), including tuning and layer latencies, and confirmed that they robustly replicated previous results from V1 (24). First, we validated that L4c neurons responded earlier than all other layers in both monkeys (47, *SI Appendix*, Fig. S3*A*). In addition, we found the same ratios of simple and complex cells in V1 in both monkeys, and these simple cells were more common in L4a/4b and L4c, similar to previous reports (24), *SI Appendix*, Fig. S3 *B* and *D*).

### WaveMAP Identifies Broad and Narrow-Waveform Clusters from Negative Spiking Units.

The shape of the extracellular action potential waveform often recorded at the soma or axon hillock of a neuron, results from ion channels on the cell membrane which control the speed and propagation of the action potential (30, 48). In monkey V1, the Kv3.1b channel, which produces rapid repolarization in narrow-spiking waveforms, is commonly found in both inhibitory (in parvalbumin+ and a fraction of calbindin+ neurons) and excitatory neurons of layer 4a, 4b, and 4c (13, 14). The waveform shape can also depend on how far the neuron is from the electrode and the morphological structure of the neuron. For example, biphasic waveforms are likely somatic, while triphasic waveforms have been associated with dendritic return currents or axons (30–33). Therefore, for each neuron in this high-density Neuropixels dataset, we selected the maximum amplitude template waveform from the channels spanning the cell, thus focusing on somatic waveforms. We then normalize the waveforms to emphasize the local features of the waveform shape that depend on activity-dependent ion channel kinetics associated with different cell types.

We analyzed the negative and positive spiking waveforms separately using our previously published WaveMAP approach (34, 37) to delineate putative cell types (*SI Appendix*, *WaveMAP Analysis*). WaveMAP uses uniform manifold approximation and projection (UMAP) on normalized waveforms to first create a high-dimensional graph, followed by Louvain clustering to delineate putative cell types. WaveMAP outperforms the traditional approaches that rely on a small number of classical features, such as trough-to-peak duration or repolarization slope, and has been used in other studies to identify candidate cell types (31, 34, 49), *SI Appendix*, Fig. S2*A*). Moreover, distributions of classical features such as trough-to-peak duration and amplitude were largely unimodal for this dataset (*SI Appendix*, Fig. S2 *C*, *E*, and *G*), and thus classical methods of cluster identification would be unsuccessful for this dataset.

**Fig. 2.**
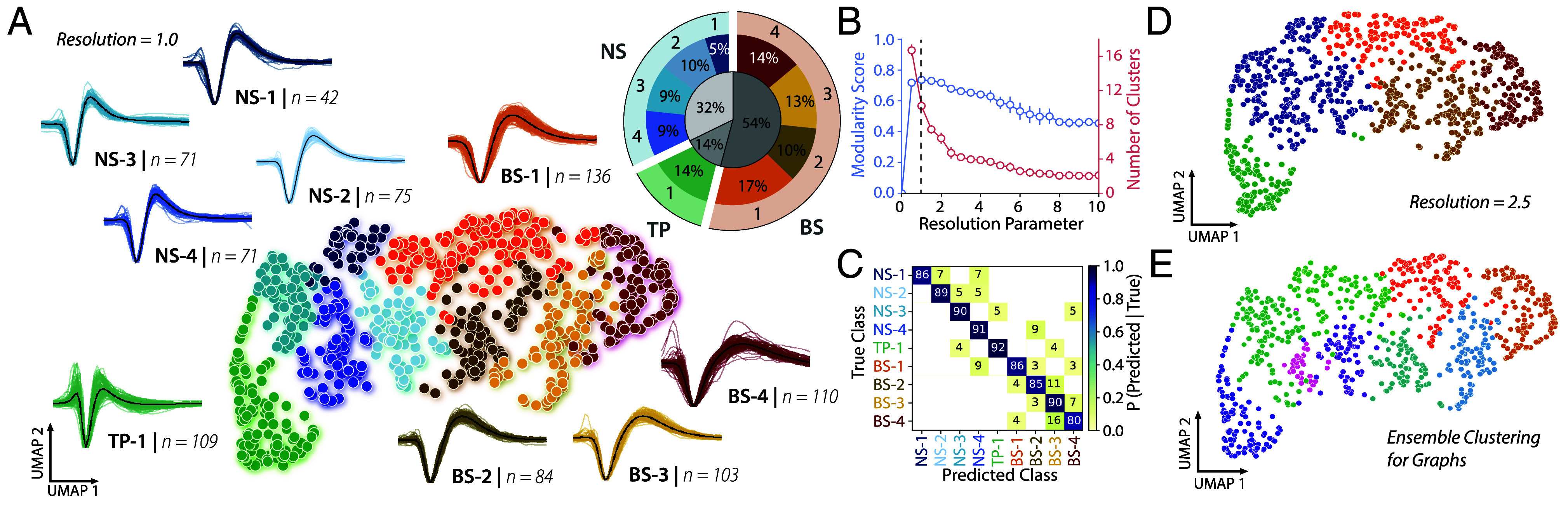
UMAP and Louvain clustering on 801 negative spiking waveforms reveals 9 candidate cell types. (*A*) Scatter plot of waveforms in UMAP space colored by Louvain cluster membership. WaveMAP parameters were set as the following: N_neighbors = 20; MIN_DIST = 0.2; RESOLUTION = 1.0. Cooler colors denote narrow-spiking clusters, and warmer colors denote broad-spiking clusters. Adjacent to each numbered cluster is shown all member waveforms and the average waveform shape (in black). Each waveform is 1.8 ms in duration. *Inset*, Population percentages by cluster. (*B*) Modularity score (in blue, on the *Left* axis) and number of clusters (in red, on the *Right* axis), as a function of the Louvain clustering resolution parameter. This plot was obtained by averaging results from WaveMAP using 25 random samples of 80% of the full dataset at each resolution parameter from 0 to 10 in 0.5 unit increments (a subset of the data was used to obtain error bars). Each data point is the mean ± std. The vertical dashed line indicates the maximization of modularity (34). (*C*) The confusion matrix of a gradient boosted decision tree classifier with five-fold cross-validation. The main diagonal shows accuracy of waveform classification for each cluster, and off diagonals show misclassification percentages (34). The average accuracy across all clusters was 88%. (*D*) Scatter plot of waveforms in UMAP space colored by Louvain cluster membership with RESOLUTION = 2.5. Conventions as in (*A*). All other WaveMAP parameters were same as in (*A*). (*E*) An alternative version of the Louvain clustering algorithm (ensemble clustering for graphs (50), requires setting no resolution parameter, and produces 9 clusters similar to our clustering at RESOLUTION = 1.0. There was good overlap between ECG and WaveMAP clusters (MARI Score: 0.62).

WaveMAP, like all machine learning algorithms, benefits from large and high-quality datasets. To improve the results of the WaveMAP clustering, we included all 801 negative spiking waveform templates, then chose the 606 neurons with visual responsiveness from this population. Fig. 2*A* shows the result of the WaveMAP analysis on 801 negative waveforms. We used a resolution parameter of 1.0 for Louvain clustering, to maximize modularity by optimizing the community “connectedness” (34), Fig. 2*B*), and found 9 clusters captured the heterogeneity of waveform shapes in this dataset (*SI Appendix*, Fig. S2 *B*, *D*, and *F*).

The cluster shapes were either narrow and triphasic (TP-1, green, Fig. 2*A*), narrow and biphasic with a sharp after hyperpolarization phase (Clusters NS1-4, cooler colors, Fig. 2*A*) or broad and biphasic with a slower after hyperpolarization phase (Clusters BS1-4, warmer colors, Fig. 2*A*). We quantified the fraction of neurons in each cluster and each general category (*Inset*). Most neurons were broad-spiking (54%). A smaller percentage of neurons had narrow, triphasic (14%) waveforms, and narrow-spiking waveforms made up the remaining sample (32%).

We also used multiple other approaches to validate the choice of resolution parameter and number of clusters. First, a classification analysis (fivsfold cross validation) found that clusters were well separated (mean accuracy of 88%) with minimal misclassification (Fig. 2*C*). Second, we used an alternative Ensemble Clustering for Graphs (ECG) approach that does not require a fixed resolution parameter and again found that the data are well explained by 9 clusters (Fig. 2*E*), with good overlap from the results of Louvain clustering (MARI Score: 0.62). Third, Louvain clustering is hierarchical. A decrease in the resolution parameter does not lead to an entirely new clustering result. Instead, some clusters are merged together while other clusters are kept intact. In our case, clusters NS1-4 merged into one narrow-spiking waveform cluster. Clusters BS-2 and BS-3 merged into another cluster, and clusters BS-1, BS-4, and TP-1 remained independent and stable (Fig. 2*D*). Thus, our choice of the resolution parameter of 1.0 ensure that we captured sufficient diversity of cell types (maximizing the modularity score), while avoiding overclustering on this dataset.

As a final check, we also tested whether the UMAP dimensions and identified clusters explained variance in functional responses. If the clusters are meaningful, then the UMAP dimensions and depth combined should explain more variance in the functional properties than just the depth at which a neuron is found. Consistent with this hypothesis, a regression analysis (*SI Appendix*, Fig. S2*H*) revealed that depth and UMAP dimensions explained more variance (9.7%, as measured by adjusted $R^{2}$) in functional properties compared to just depth alone (3.0%). Similarly, depth and candidate cell type explained more variance (8.4%) than depth alone. Finally, these effects were not a trivial artifact of just signal-to-noise ratio as it only explained a small fraction of the variance (0.4%) and far less than the variance explained by UMAP and depth or candidate cell type and depth. Together, the clustering analyses suggest that nine clusters balance the diversity of candidate cell types while minimizing overclustering of this dataset.

### Narrow-Spiking Waveforms Are More Likely in Granular Layers, and More Common than PV Neurons.

Anatomical studies suggest that potassium channels (e.g., in the Kv3 family) that lead to rapid repolarization and narrow-spiking waveforms are more prevalent in L4a, L4b, and L4c than in other layers of V1 (14), and although commonly associated with fast-spiking PV cells, are found in both excitatory and inhibitory neurons (13, 51, 52). These Kv3 positive, excitatory neurons are largely restricted to layers 4b and 4c (14, 53). A prediction from these studies is that 1) narrow-spiking neurons should be more common in L4a/4b and L4c compared to other layers, 2) they should be more common than PV neurons, and 3) there should be a subpopulation largely restricted to L4a/4b and L4c with narrow-spiking waveforms.

Overall, observing the laminar distributions of all clusters, we found a significant difference in their mean depths (F(8,597) = 9.64, $P=1.3\times 10^{-12}$). To further test these predictions, we performed three other analyses.

**Fig. 3.**
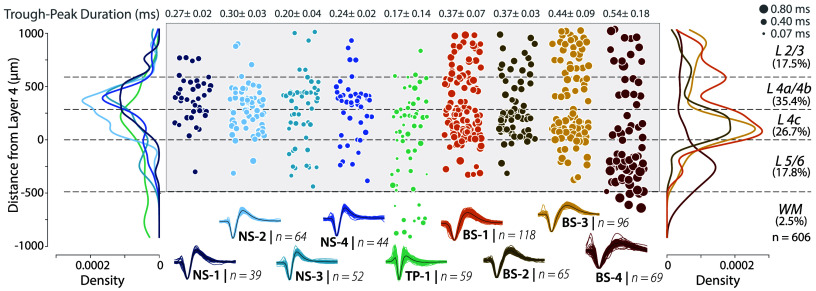
Narrow-spiking waveforms are more common in L4a/4b and L4c. *Center* panel (shaded in light gray) shows a scatter plot of laminar location for neurons in each of the clusters. The mean and the std of the trough-to-peak duration of the waveforms are indicated at the *Top* of each plot. Dashed lines depict layer boundaries identified from CSD and histology. Size of the markers indicates trough-to-peak duration. *Left* and *Right* panels show the density distributions for the cell populations. Percent of units in each layer shown in parentheses (shown on the *Right*).

First, we assessed the laminar distribution of NS1-4 clusters identified from our recordings (Fig. 3), and found that all narrow-spiking classes were more common in L4c and L4a/4b compared to other cortical layers (Fig. 3, *Left* panel, density distributions, *SI Appendix*, Fig. S4 *A* and *B* and Table S1). 77% ± 2.97% (mean ± SEM) of NS neurons were found in L4a/4b and L4c, whereas only 12.5% were found in L2/3 and 10% were found in L5/6 ($\chi^{2}$ (2, 199) = 173.97, P<0.0001, *SI Appendix*, Fig. S4*B*). This percentage was significantly larger than expected from a shuffle control that randomly reassigned neurons to L4a/4b or L4c while preserving the total number in each category (Shuffle test, P<0.002). Additionally, the mean distance from layer 4 for all narrow-spiking neurons was 304 μm (95% CI: 266.28, 342.04 μm) and well within the boundaries of L4a/4b and 4c (0 μm to 588.8 μm). These analyses suggest that the narrow-spiking neurons are common in L4a/4b and 4c compared to other layers, supporting a prediction from an anatomical study (14).

Second, we compared the percentage of narrow-spiking neurons, composed of NS1-4, in the final dataset (606 units) with the percentage of PV neurons expected from anatomical studies of V1. Our estimate of narrow-spiking neurons was 32% ± 1.6% (mean ± SEM), which was significantly above the fraction of PV neurons reported in the literature (5 to 10%, CIs do not overlap) (14, 52). Together, these results are consistent with our predictions from anatomy that narrow-spiking neurons should be more common in L4a/4b and L4c and likely observed in both excitatory and inhibitory neurons.

Third, we assessed the laminar distribution of each of the NS1-4 subclasses to examine if a subpopulation was largely restricted to L4a/4b and L4c (*SI Appendix*, Fig. S4 *C* and *D*), which may account for the excitatory Kv3 positive neurons previously found in anatomical studies (14). Consistent with the structural prediction, 86.4% ($\chi^{2}$ (2, 103) = 131.33, P<0.0001) of NS-1 and NS-2 subclasses was only observed in L4a/4b and L4c and far less prevalent in L2/3 (9.7%) and L5/6 (3.9%). In contrast, although NS-3 and NS-4 were strongly concentrated in L4a/4b and L4c (67.7%, $\chi^{2}$ (2, 96) = 52.94, P<0.0001), they were also found in L2/3 (10.47%) and L5/6 (21.9%). These analyses show a localization of narrow-spiking neurons in L4 within the V1 laminar microcircuitry.

### The Largest Amplitude Neurons in V1 Are Located in Layer 4b and Show Strong Direction Selectivity.

Anatomical studies of V1 suggest that a subpopulation of stellate neurons in layer 4b with dense dendritic arborization and some of the largest soma sizes project to MT (15, 54), and thick stripes of V2 (19). In parallel, an antidromic stimulation study identified that neurons that project from V1 to MT are strongly direction selective (26). Together, these studies predict a subpopulation of neurons strongly localized to layer 4b with large amplitudes and strong direction selectivity.

We tested this prediction by examining the relationship between spike amplitude of the cell classes and their direction selectivity. We used spike amplitude as an estimate of cell size based on observations that it is related inversely to input resistance with larger neurons having smaller resistance and positively correlated to the size of the soma and the dendritic arbor (55). If there is a population of putative MT (and likely V2) projecting neurons with large soma and extensive dendritic arbors, we should expect that this cluster should 1) have the largest spike amplitudes, 2) be more likely in 4b, and 3) be selective for direction. Note, there is no guarantee that there is a uniform distance from each unit to the recording probe. However, such an effect would lead to a more uniform distribution of cell sizes as a function of cluster.

**Fig. 4.**
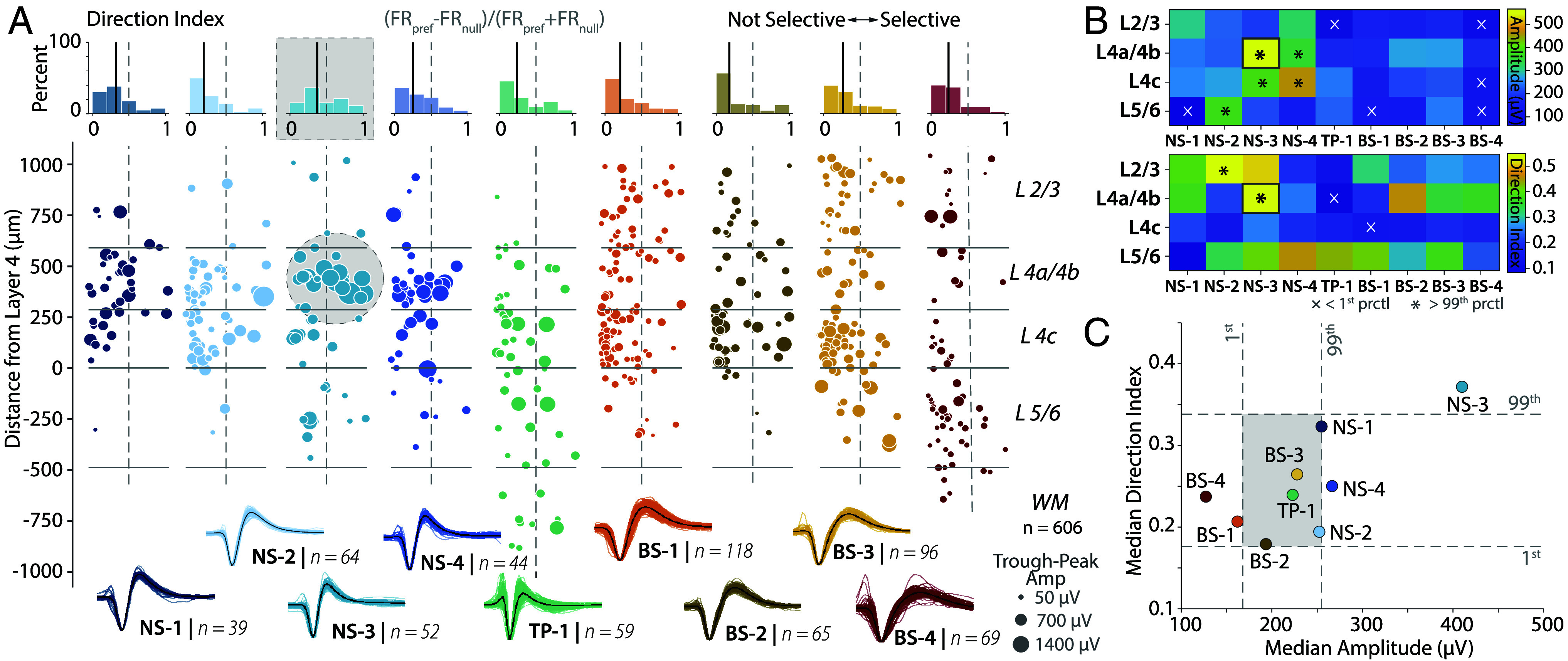
The NS-3 cluster shows strong direction selectivity and high amplitude. (*A*) *Center*, A scatter plot of direction selectivity (x-axis) vs. depth (y-axis) for visually responsive neurons in each of the clusters. The points are each colored by the cluster. The marker size for neurons within each cluster is scaled by the nonnormalized amplitude of each unit (μV). Solid lines depict layer boundaries identified from CSD and histology. *Top*, Histograms of the direction selectivity index. The bold line indicates the median direction index for each cluster. The vertical dashed center lines show the boundary between not selective (*Left* of *Center*) and selective (*Right* of *Center*). (*B*) *Top*, Heatmap of nonnormalized trough-to-peak amplitudes as a function of cluster and layer. *Bottom*, Heatmap of direction index as a function of cluster and layer. For both, X indicates the median is < 1st percentile, * indicates > 99th percentile, for the shuffled distributions estimated from 1,000 shuffles of layer and cluster. (*C*) Plot of median direction index and cluster relative amplitude (μV) for each of the clusters. Dashed lines indicate 1st and 99th percentile for the shuffled distributions estimated from 1,000 shuffles of direction index and amplitude. Only the NS-3 class (in L4a/4b from *B*) has median amplitude and direction index significantly greater than the shuffled distributions.

We tested this prediction using the following analysis. Fig. 4*A* shows a scatter plot of direction selectivity as a function of candidate cell type and layer. Amplitude varied widely as a function of cell type and layer. We found amplitude (F(8,598) = 20.85, $P=5.5\times 10^{-28}$) and direction selectivity (F(8,598) = 2.47, P=0.012) to be significantly different among all clusters. We also observed a modest positive correlation between amplitude and direction selectivity (partial Pearson correlation controlling for SNR, Pearson’s r = 0.21, P<0.001). Moreover, consistent with our predictions, the large amplitude neurons were more common in layer 4b and also strongly direction selective (Fig. 4*A*, see gray circle).

We quantified these qualitative patterns by measuring the median amplitude of the waveforms as a function of cluster and layer (Fig. 4*B*). Consistent with both the qualitative picture obtained from (Fig. 4*A*) and our prediction, the largest amplitude neurons were found in layer 4b. If our hypothesis that these large neurons in layer 4b project to MT is correct, then they should be strongly direction selective. Consistent with our prediction, we found that the NS-3 cluster, largely localized to layer 4b, had the largest amplitude and the strongest direction selectivity (26), Fig. 4 *B* and *C*). Only for the NS-3 class were direction selectivity and amplitude significantly different from the shuffled distribution (Shuffle test, P<0.002, Fig. 4 *B* and *C*).

Analysis of the relationship between amplitude and direction selectivity also provided insights into broad-spiking neurons. Clusters BS-4 and BS-1 have the smallest spike amplitudes and were significantly different from a shuffled distribution (Fig. 4*C*). Cluster BS-4 is localized to L5/6, whereas cluster BS-1 is most common in L4c (*SI Appendix*, Fig. S4*D*). These findings are also consistent with anatomical observations: Electron microscopy studies suggest that most pyramidal neurons in layer 5 of V1 are small (56). Stellate neurons in L4c can range from small to large. However, the average soma size is smaller in the 4c layers compared to L2/3 and layer 4b (56). Finally, clusters BS-2 and BS-3 are larger in amplitude and more concentrated in L4c and L2/3. However, cluster BS-2 has significantly less direction selectivity than cluster BS-3.

While these results are contingent on links between voltage amplitude and neuron size (38), these results are evidence for a functional population of neurons in layer 4b with large spike amplitudes and strong direction selectivity (15).

### A Narrow-Spiking Cluster, NS-1, Is Localized to Layer 4b and Has “Bursting”-Like Properties.

In vivo and in vitro studies of V1 predict neural populations with bursting activity in V1 (21, 22, 57, 58). In vitro studies in the cat predict existence of narrow-spiking neurons with strong bursting activity in layers 2 to 4 (21), and broad-spiking neurons with bursting activity in all layers. Furthermore, one in vivo study suggests that bursty, narrow-spiking neurons should be strongly orientation selective (22). One signature of “bursting” is peaky interspike interval (ISI) distributions. Collectively, these studies predict narrow-spiking clusters in layers 2 to 4 with strongly peaked ISI distributions and strong orientation selectivity.

**Fig. 5.**
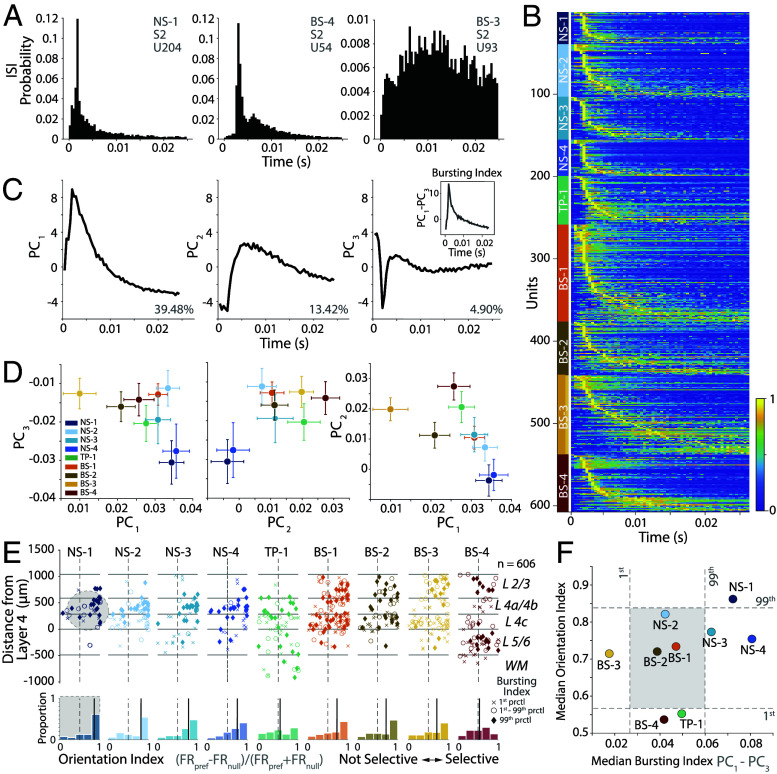
A Narrow-spiking, bursty population localized to V1 L4a/4b and L4c is strongly orientation selective. (*A*) Interspike interval histogram normalized to relative probability of three example units from cluster NS-1, BS-4, and BS-3. (*B*) Heatmap of normalized ISI distributions of all visually responsive units, sorted by ISI peak, and by cluster. (*C*) First three PCs of the ISI distributions. *Inset*, The difference between $PC_{1}$ and $PC_{3}$ emphasize the “peaked” shape of an ISI distribution consistent with bursting. (*D*) *Left*, Scatter plot of mean cluster values for $PC_{1}$ and $PC_{3}$. *Middle*, Scatter plot of mean cluster values for $PC_{2}$ and $PC_{3}$. *Right*, Scatter plot of mean cluster values for $PC_{1}$ and $PC_{2}$. Errorbars indicate SEM. (*E*) *Top*, Scatter plot of orientation selectivity (x-axis) vs. laminar location (y-axis) for neurons in each of the clusters. X marker indicates neurons with a bursting index < 1st percentile, O marker indicates between 1st to 99th percentile, and a filled diamond indicates > 99th percentile shuffled distribution (estimated from 1,000 shuffles), showing the most “peaked” ISI distributions. Solid lines depict layer boundaries. *Bottom*, Panel below the scatter plots shows histograms of the orientation selectivity index. The bold line indicates the median orientation index for each cluster. The vertical dashed center lines show the boundary between not selective (*Left* of *Center*) and selective (*Right* of *Center*). (*F*) Scatter plot of median orientation index and median bursting index (difference $PC_{1}$ and $PC_{3}$) per cluster for each of the clusters along with the 1st and 99th percentile for the shuffled distributions estimated from 1,000 shuffles. Cluster NS-1 has significantly different orientation selectivity and bursting from the shuffled distributions.

Fig. 5*A* shows the ISI probability distribution for one narrow-spiking and two broad-spiking example neurons in our dataset, showing peaked vs. wide ISI distributions. We calculated the normalized ISI distributions for visually responsive neurons, sorted by the ISI peak and separated by cluster (Fig. 5*B* and *SI Appendix*, *Interspike Interval*). This analysis suggests that the neural population in V1 is heterogeneous and includes neurons with both peaked and wide ISI distributions. However, the narrow-spiking clusters are more likely to have peaky ISI distributions compared to some of the broader-spiking clusters (e.g., NS-1, compared to BS-3).

To better understand this heterogeneity, we performed PCA on the normalized ISI distributions. We first examined the components of the ISI distribution and found that $PC_{3}$ had a strong dip for short ISIs. Thus, ISI distributions that are more bursting are likely to have a strong negative loading on this principal component.

In contrast, both $PC_{1}$ and $PC_{2}$ were more similar to ISI distributions expected from regular spiking activity with refractory periods (Fig. 5*C*). If the in vivo prediction of a population of narrow-spiking, bursting neurons with high orientation selectivity in V1 is correct (22), then we expect some narrow-spiking clusters with strong negative loadings on $PC_{3}$ and positive loadings on $PC_{1}$. Consistent with this prediction, both NS-1 and NS-4 clusters had negative loadings on $PC_{3}$ and positive loadings on $PC_{1}$ suggesting that these units were more likely to be bursting (Fig. 5*D*). In contrast, BS-3 had minimal to no loading on $PC_{3}$ suggesting that these neurons were more likely to be regular spiking.

We calculated a bursting index, defined as the difference in loadings between $PC_{1}$ and $PC_{3}$ for each of the clusters to capture this trend (*SI Appendix*, *Interspike Interval*). We examined relationships between orientation selectivity and the bursting index to test the hypothesis that the bursting narrow-spiking neurons are strongly orientation selective (22). Fig. 5*E* shows a scatter plot of the orientation selectivity of neurons for each of the clusters, with the dot size a function of the burst index for each neuron. Overall, we found a modest relationship between bursting index and orientation selectivity (partial correlation controlling for SNR, Pearson’s r = 0.1250, P<0.01). Moreover, some clusters were more likely to be strongly bursting and orientation selective. NS-1 had a strongly peaked distribution for orientation selectivity and higher burst indices consistent with ref. 22. In contrast, NS-4 had broader distribution of orientation selectivity while being likely to have a ISI distribution consistent with bursting. Similarly, NS-2 showed some orientation selectivity but was less likely to show peaked ISIs that are consistent with bursting. Note that the mean firing rate of all of these cells was largely similar (∼20 spikes/s, *SI Appendix*, Fig. S3 *A* and *C*), suggesting that these ISI distributions are the result of rapid “burst” like patterns of spiking in response to the sensory stimulus, and not just a trivial effect of the firing rate.

Finally, we tested the relationship between orientation and bursting. A single factor ANOVA found orientation selectivity (F(8,598) = 4.23, $P=5.9\times 10^{-28}$), and bursting index (F(8,598) = 7.94, $P=3.4\times 10^{-10}$) to be significantly different among the clusters. Moreover, the median bursting index and the median orientation selectivity for NS-1 was significantly different from both shuffled distributions (Shuffle test, P<0.002, Fig. 5*F*). In contrast, BS-3 had the lowest bursting index suggesting that this cluster was more likely to be regular spiking (Shuffle test, P<0.002).

These results suggest that bursting activity is observed in all clusters. However, a subpopulation of narrow-spiking neurons in V1 (NS-1), on average are 1) more likely to have peaky ISI distributions consistent with bursting, 2) localized to layers 2 to 4, and 3) orientation selective. These results are consistent with predictions of ex vivo electrophysiological studies of cat V1 (21), and provide laminar localization of bursting narrow-spiking neurons in V1 (22).

### Narrow Clusters Have Distinct Multichannel Waveforms Consistent with Specific Morphologies.

Neuropixels measure the multichannel waveform for a neuron (*SI Appendix*, *Multichannel Profile*). Modeling and experimental studies suggest that the multichannel waveform is at least in part influenced by the morphology of the cell (59, 60). In this section, we examine whether the multichannel waveforms of these neuronal clusters are consistent with descriptions of the morphology of various cell types reported in V1 (59, 60).

In Fig. 5, we identified that the NS-1 cluster was strongly bursting and orientation selective. These narrow-spiking, bursting cells are thought to be excitatory and pyramidal in nature (21). A recent Neuropixels study of mouse V1 (60) suggested that pyramidal neurons in V1 show evidence of strong unidirectional propagation of action potentials toward the dendrites whereas inhibitory and spiny stellate neurons are more likely to have symmetric waveforms. Thus, if the NS-1 cluster is an excitatory pyramidal cell type, it should show unidirectional propagation of action potentials. Similarly, the NS-3 cluster had large amplitude and high direction selectivity, which were remarkably consistent with anatomical findings about neurons that project from V1 to MT (15). An additional prediction from this study is that the neurons that project from V1 to MT are likely to be stellate in nature (15). Again, modeling studies (59) suggest that nonpyramidal neurons lacking large apical dendrites and instead possessing dense arborization around the soma (e.g., spiny stellate cells in layers 4a, 4b, and 4c) are more likely to have symmetric multichannel waveforms.

We first tested the prediction that NS-1 is an excitatory pyramidal neuron with a strong unidirectional multichannel waveform (60). Second, we assessed whether the multichannel waveform of the NS-3 cluster was more likely to be symmetric consistent with a stellate morphology (59).

**Fig. 6.**
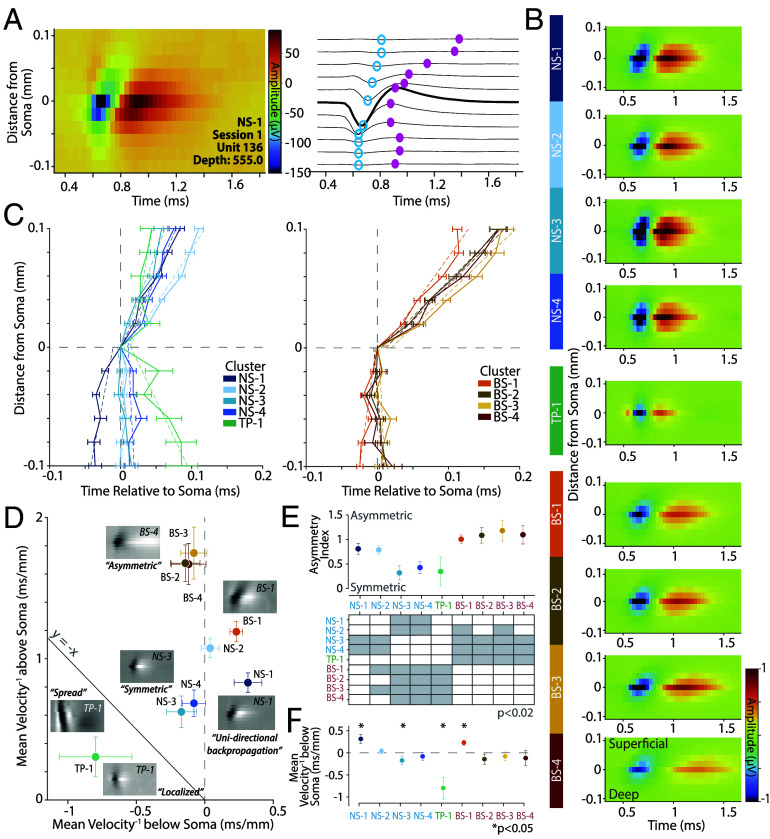
Multichannel waveforms can help better delineate cell types. (*A*) *Left*, A heatmap of the maximum amplitude channel, assumed to be the position of the soma, and five channels above and below. The unit is identified by cluster, session, unit ID, and depth. *Right*, A time-series representation of each channel. The largest amplitude waveform is in bold. The time of waveform troughs (open blue points) and peaks (closed magenta points) of each channel are indicated. (*B*) Median multichannel extracellular waveforms of all neurons per cluster. The colorbar shows the scale standardized for all clusters between −1 and 1 μv. (*C*) The trough trajectory, or the mean time of waveform trough at each channel location above and below soma (aligned to y-axis = 0, removing outliers using 95% CIs) ± SEM. Velocities above and below soma and are estimated separately by linear regression (slope of the dashed line). *Left*, Narrow and triphasic clusters. *Right*, Broad clusters. (*D*) Mean trough propagation velocity above soma vs. below soma for each cluster. Clusters with positive slopes both above and below the soma are located on the *Right* of x-axis = 0. Clusters which show positive slopes above and negative below the soma are located on the *Left* of x-axis = 0. Errorbars are std calculated from bootstrapped data (500 resamples). (*E*) *Top*, Asymmetry index of each cluster (*SI Appendix*, *Asymmetry Index*). This is the absolute value of the distance of each cluster’s propagation velocity above and below the soma, from the y = −x line. *Bottom*, Errorbars are bootstrap SE (500 resamples). Pairwise comparisons between clusters were performed using independent bootstrap tests (500 resamples per comparison). The heatmap indicates statistically significant differences (98% bootstrap CI excludes zero, P<0.02). (*F*) The mean slope of the propagation velocity below the soma was plotted per cluster. Errorbars are bootstrap SE (500 resamples). Independent bootstrap tests were done to compare each slope to 0 as a measure of the propagation velocity direction with asterisks indicating significance (95% bootstrap CI excludes zero, P<0.05).

The *Left* panel of Fig. 6*A* shows the multichannel profile of an example neuron from NS-1 (with a narrow-spiking waveform) centered on the maximum amplitude channel which is assumed to be the channel closest to the soma (30). The *Right* panel of the same figure shows the waveform as a function of time from each of the corresponding channels (*SI Appendix*, *Propagation Velocities*). Channels above the soma showed progressively delayed troughs compared to the somatic waveform (bold trace in Fig. 6*A*) suggesting upward action potential propagation toward the pia.

Fig. 6*B* shows the average multichannel profile of the nine clusters. BS1-4 show asymmetric waveforms with strong propagation of action potentials away from the soma toward the pia. In contrast, NS-3 and NS-4 shows symmetric multichannel waveforms with minimal action-potential propagation away from the soma, whereas NS-1 propagates both to and away from the soma in the same direction. These visualizations are consistent with the predictions that NS-1 is more likely to be pyramidal in nature, whereas NS-3 is more likely to be stellate in nature.

We categorized the trough propagation trajectories in Fig. 6*C* by plotting the time of the trough relative to the distance from the soma (e.g., blue dots in Fig. 6*A*). The *Left* plot shows the narrow-spiking and triphasic clusters and the *Right* plot shows the broad-spiking clusters. On average, clusters NS-2 and BS2-4 show an asymmetric profile consistent with propagation above the soma toward the pia as predicted. In contrast, cluster NS-1 and BS-1 demonstrated *unidirectional propagation* consistent with morphologies of pyramidal neurons, whereas both NS-3 and NS-4 were far *more symmetric* in their multichannel profiles with only minimal propagation of action potentials away from the soma.

Fig. 6*D* shows a scatter plot of the mean propagation velocity above and below the soma for each cluster. We found that the unidirectional and bidirectional clusters fell into different quadrants on this plot. BS2-4 were largely asymmetric and had minimal propagation below the soma but strong propagation above the soma. NS-3 and NS-4 had more symmetric propagation above and below the soma (closer to the y = −x line). Finally, NS-1 and BS-1 showed upward propagation both above and below the soma.

We calculated an asymmetry index (Fig. 6*E* and *SI Appendix*, *Asymmetry Index*), and also examined the velocity below the soma (Fig. 6*F*). Clusters NS-1, NS-2, and clusters BS1-4 had a large asymmetry index (98% bootstrap CI excludes zero, P<0.02) suggesting uneven propagation of the action potential away from the soma. In contrast, clusters NS-3, NS-4, and TP-1 were strongly symmetric, suggesting propagation of the action potential both above and below the soma. Symmetric action potentials are predicted for dense morphologies such as those observed for stellate neurons suggesting that NS-3 and NS-4 might possess stellate-like morphologies. Finally, NS-1 and BS-1 were the only clusters with a significant positive slope below the soma (95% bootstrap CI excludes zero, P<0.05) showing unidirectional propagation of the action potential (Fig. 6*F* and *SI Appendix*, Fig. S5 *D*–*G*).

Collectively, these results are consistent with our hypotheses that NS-1 cluster is likely to be an excitatory neuron population. In contrast, the multichannel waveform of NS-3 (and NS-4) is more consistent with dense dendritic morphologies around the soma, which is expected of nonpyramidal stellate neurons and thus consistent with anatomical descriptions of neurons that project from V1 to MT (15).

### Interlaminar Information Flow Is Cluster Specific.

Our final analysis was to understand the flow of information within V1 laminar microcircuitry. To this end, we calculated the normalized, and jitter-corrected cross-correlograms (CCGs) of all visually responsive neurons (*SI Appendix*, *Cross-Correlations and Connectivity*). We then labeled significant CCG pairs between neurons in different WaveMAP clusters (n = 6,261) as those for which peak CCG was 7 SD above the noise distribution (*Methods*). This identified whether the reference cell in a cluster led (peak lag > 0 ms) or lagged (peak lag < 0 ms) the target cell in a cluster.

**Fig. 7.**
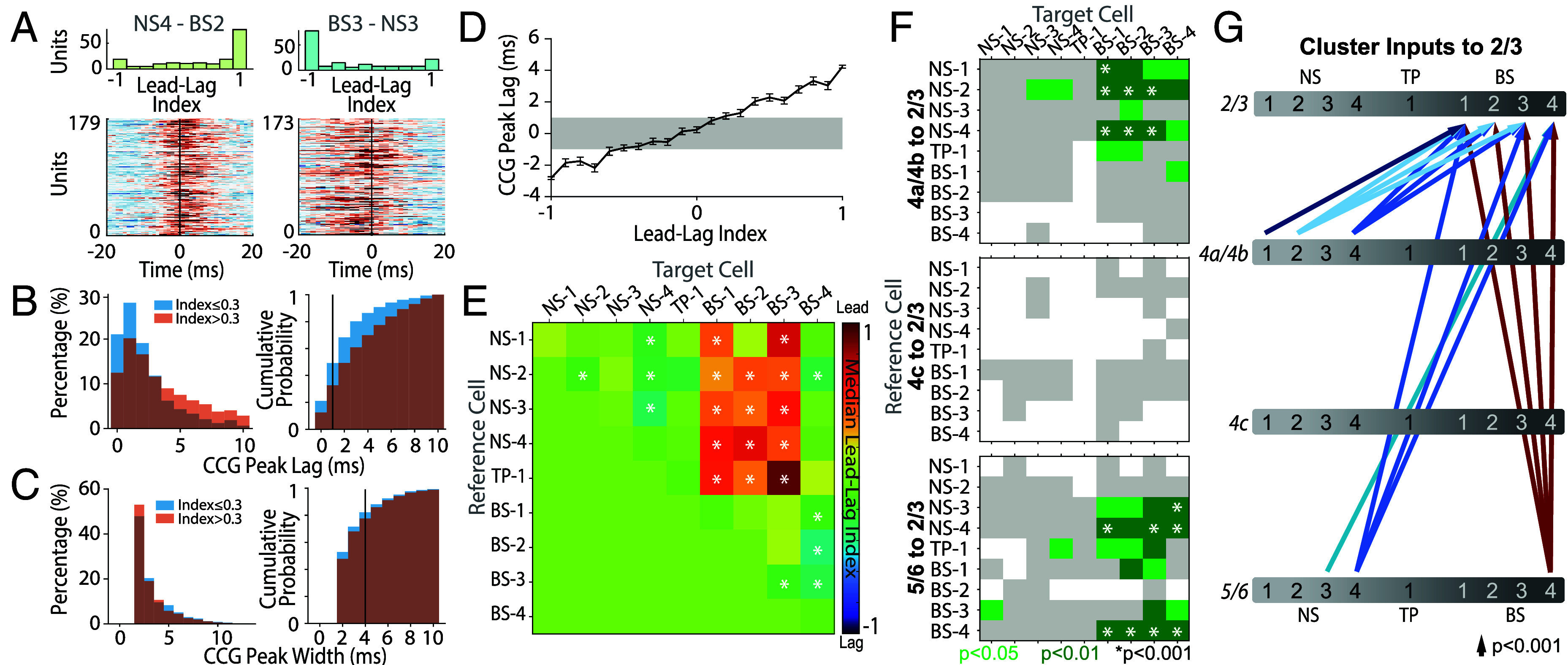
Cross-correlations reveal V1 laminar connectivity between putative cell types. (*A*) Example cross-correlograms (CCG) between clusters. *Top*, Histograms of CCG asymmetry (lead-lag index). *Bottom*, Heatmap of pairwise CCGs. *Left*, Cluster NS-4 leading BS-2. *Right*, Cluster BS-3 lagging NS-3. (*B*) Distribution of all CCG peak lags separated by lead–lag index of 0.3. CCGs with peak lag < 1 ms may receive common input. (*C*) Distribution of all CCG peak widths separated by lead–lag index of 0.3. CCGs with peak width > 4 ms may receive common input. (*D*) CCG peak lag plotted against the lead–lag index for all CCGs among the clusters. Errorbars show ± SEM. Gray shadow indicates CCGs that may receive common input. (*E*) Heatmap of pairwise cross-correlation median lead–lag indices between cluster. The reference cell (*Left*) cluster interacts with the target cell (*Bottom*). If the reference leads the target, the color is red, if it lags behind the target, the color is blue. * indicates Wilcoxon signed rank sum test significance (P<0.05). (*F*) Heatmaps showing significant lead–lag indices between clusters in layer 4b which lead clusters in layer 2/3 (*Top*), clusters in L4c which lead clusters in layer 2/3 (*Middle*), and clusters in layer 5/6 which lead clusters in layer 2/3 (*Bottom*). Gray indicates insignificant CCGs, and * indicates Wilcoxon signed rank sum test significance (P<0.001). (*G*) Network diagram showing significant interactions between clusters in all layers and clusters in layer 2/3. Arrows indicate Wilcoxon signed rank sum test significance (P<0.001) and direction, from the reference to the target. CCGs from M2 are underrepresented in both (*F* and *G*) due to the low number of L2/3 neurons recorded.

We calculated a lead–lag index for each significant CCG by subtracting the CCG values on the right side (lead) of time lag 0 from CCG values on the left side (lag) of time lag 0, and then normalized by their sum (*SI Appendix*, *Lead–Lag Index*). Small absolute values of the lead–lag index suggest that the neuronal pair receive common input and thus exhibits a center-peaked CCG. In contrast, large absolute values (close to 1) indicate temporal asymmetry consistent with excitatory connections (61). While the correspondence between the lead–lag index and the CCG peak offset can vary for individual pairs, the pattern is more evident at the population level. Of the significant CCGs between neurons in different clusters, a minority (22%) had a lead–lag index ≤ 0.3 perhaps reflecting common inputs. However, the majority (78%) of the CCGs had a lead–lag index > 0.3. For all CCGs with lead–lag index > 0.3, median peak lag was 3 ms and median peak width was 2 ms (Fig. 7 *B* and *C*), suggesting they exhibit offset peaks. The CCGs with lead–lag index > 0.3 also showed a significantly different peak lag (Wilcoxon rank sum test, P<0.001) and a significantly different peak width (Wilcoxon rank sum test, P<0.001) from the CCGs with lead–lag index ≤ 0.3. When observing all lead–lag indices with respect to their peak lags (Fig. 7*D*), the mean peak lags of indices near 1 and -1 are outside ± 1 ms of zero. Together these analyses suggest that the majority of CCGs involved cell to cell interactions.

Fig. 7*A* shows two examples of lead–lag interactions between clusters, where NS-4 leads the BS-2 cluster (*Left* panel) and BS-3 lags NS-3 (*Right* panel). These examples suggest that the population analysis of CCGs show connectivity trends between WaveMAP clusters. We summarized all the pairwise relationships using a heatmap and assessed the significance of this index by using a sign-rank test (Fig. 7*E* and *SI Appendix*, *Lead–Lag Index*). These analyses revealed that all narrow-spiking clusters lead the broad-spiking clusters BS1-3, with the exception of BS-4. This BS-4 cluster also led all other broad-spiking clusters, as well as cluster NS-2. Moreover, within the narrow-spiking clusters, NS-4 noticeably led NS-1, NS-2, and NS-3 clusters.

We further quantified these lead–lag relationships between different pairs of candidate cell types and layer. Fig. 7*F* shows the significant CCG pairs of neurons between clusters and layers with respect to layer 2/3 (CCGs from M2 are underrepresented here due to low neuron yield). For example, cluster NS-4 (the reference cell) in L4a/4b has a significant (Wilcoxon rank sum test, P<0.001) lead–lag index > 0.3 with cluster BS-1 (the target cell) in L2/3. This analysis suggests a general trend of narrow-spiking clusters (NS-1, NS-2, NS-3, NS-4, and TP-1) in layer 4 leading broad-spiking clusters (BS-1, BS-2, and BS-3) in L2/3 with the exception of BS-4, largely localized to L5/6, which also leads the other broad clusters (Fig. 7*G*). Together, significant interactions between clusters and among layers suggest that there is a flow of information from infragranular and granular layers to supragranular layers, consistent with a feedforward model of local circuitry (6). Interestingly, although L4c shows an earlier response to stimuli than other layers (*SI Appendix*, Fig. S3*A*), it is not significant in this CCG analysis.

## Discussion

The goal of this study was to better understand the link between laminar microcircuitry in monkey V1 and visual function. To this end, we reanalyzed Neuropixels recordings across layers of V1 of two anesthetized rhesus macaques (28). We applied WaveMAP (31, 34, 37, 49) on the waveforms to identify candidate cell classes. These cell classes exhibited distinct functional properties with respect to their laminar organization, tuning properties, bursting patterns, and multichannel waveforms.

We used CSD in combination with histological estimates to determine the layer boundaries of our recordings and thereby identify the layers in which we found different candidate cell types. Such analyses revealed that narrow-spiking neurons were approximately 30% of the neurons that we recorded in V1, and >75% of these neurons were found in L4a/4b, and L4c. These findings are consistent with reports from anatomical studies that 80% of Kv3 and especially Kv3.1b positive neurons are found in L4a, L4b, and L4c of V1 (14).

However, this ∼30% proportion is larger than what is expected from anatomical studies. Only ∼7% of all neurons in a V1 column are Kv3.1b positive and thus likely to be narrow-spiking (14). This discrepancy could emerge for a few reasons. First, WaveMAP is an unsupervised clustering method: It is entirely possible that we overestimated the fraction of narrow-spiking neurons. Even the narrow-spiking neurons that we identified had a range of trough-to-peak durations (0.27 ms and 0.30 ms, for NS-1 and NS-2 classes, and 0.2 to 0.24 ms for NS-3 and NS-4 classes). One possibility is that the Kv3.1b positive units are only the NS-3 and NS-4 classes which had trough-to-peak durations closer to 0.2 ms, in which case, the numbers would be more similar to what is observed in anatomical and in vitro studies (14, 62, 63). Second, that particular study (14) only identified the number of Kv3.1b positive neurons of V1 compared to the overall number of cells. However, other potassium channel types of the Kv3 family (such as Kv3.2), can also facilitate narrow-spiking and are known to be expressed in V1 (13), and other brain areas of primates (64). Further assessment of the expression levels of these voltage-gated potassium channels and sodium channels that can confer fast spiking will help better estimate the population of fast-spiking cells in V1, which in turn will inform physiological studies (65). Third, the fraction of any given cell type is active during in vivo recordings is still unknown (66). Recordings in the auditory cortex of anesthetized rats suggest that input and feedforward layers are more active during isofluorane anesthesia compared to neurons in feedback layers (67), which could lead to the enrichment of narrow-spiking neurons in our dataset.

In layer 4b, we identified large-amplitude neurons with strong direction selectivity and multichannel waveforms consistent with stellate morphology. We speculate that these are the V1 neurons that project to MT (26). These neurons had narrow waveforms with a trough-to-peak duration of 0.2 ms, suggesting expression of fast-spiking potassium or sodium channels. Fast-spiking confers three potential advantages for motion processing: 1) sustained high firing rates, 2) greater dynamic range for encoding stimulus strength, and 3) enhanced temporal precision for tracking rapid motion. Intriguingly, anatomical studies of V1 identified a Kv3-immunoreactive neuronal population with large, elongated cell bodies that the authors suggested were likely “spiny stellate cells with horizontally extended dendritic fields in layer 4b” (13). However, due to incomplete staining of cell arbors, the authors could not conclusively classify these cells as stellate. Combining experiments that use tracers (e.g., nontoxic rabies virus) to identify neurons in V1 that project to MT (15) with approaches for examining ion channel expression could help test this prediction more rigorously (14).

We found that ISI distributions of the recorded cell classes were highly diverse. Some neurons demonstrated classical broad ISI distributions with a slow decay consistent with a Poisson process and a refractory period. However, many other neurons showed ISI distributions that had a strong peak within 10 ms. The typical assumption is that cortical neurons are largely Poisson in nature (68). However, our results suggest that even at the earliest stages of visual processing, many neurons can show ISI distributions that are not fully consistent with the Poisson assumption. We have interpreted these neurons with peaky ISI distributions as “bursting.” Our results are consistent with the findings that most of the neurons in area V1 and MT burst in response to a sensory stimulus (58, 69). Our results also reaffirm reports of bursting in anesthetized monkey V1 (57), and more recent studies suggesting the presence of narrow-spiking, bursting, excitatory neurons in awake monkey V1 (22). A key advance of this study over these previous studies is the laminar location of these neurons which showed that superficial layers and in L4a/4b and L4c are more likely to burst than neurons in deeper layers. One caveat to these findings of bursting is that we used isoflurane anesthesia, which could potentially alter the network properties such as neurotransmitter release and impact interspike intervals in these neurons (70–72) and so our findings need to be further validated in awake animals.

A key advantage of Neuropixels is the ability to densely samples neurons in an area in vivo. In V1, cells in the granular layers, especially L4c, have small cell bodies. Thus, Neuropixels might provide increased access to these cells, which may be undersampled in more traditional linear probes such as the V-probe or single electrodes. Another advantage of Neuropixels is that they record the same neuron across multiple channels and thus provide a detailed spatiotemporal profile of the extracellular waveform for each neuron. Guided by previous efforts (59, 60), we analyzed how the waveforms vary across electrodes and derived various metrics including the spread and propagation velocity for each of these clusters. These metrics potentially provide additional information to separate putative cell classes and understand how the morphology of a neuron might affect high-density extracellular recordings. For instance, at least for the mouse, clustering on multiple modalities such as interspike-interval, waveform shape, autocorrelogram, and features enables better separation of cell classes that are closer to ground-truth (73).

Analyses of the multichannel waveform also helped illuminate the diversity within the cell clusters (*SI Appendix*, Fig. S5 *A*–*C*). We showed that NS-1, a candidate cell class with ISI distributions consistent with previous reports of bursting, had strong unidirectional propagation toward the soma suggesting a particular morphology for these neurons. In contrast, NS-3 and NS-4 clusters had strong symmetric spatiotemporal profiles around the soma suggesting that their morphology was different from the NS-1 cluster and more consistent with dense local dendritic arborization. The NS-3 and NS-4 clusters also had the largest spiking amplitudes. Modeling studies suggest that dense local dendritic arborization exerts strong effects on the amplitude of the extracellular waveform but minimal to no effects on the shape of the waveform (59). We also found that the BS-1 cluster had multichannel properties similar to those observed for NS-1, with a near unidirectional propagation away from the soma. However, the BS-1 cluster also had the smallest spike amplitude and did not share the laminar compartments nor functional properties of NS-1. In contrast, the multichannel waveforms of BS clusters 2-4 propagated away from the soma toward the pia and had minimal to no propagation toward the white matter. BS2-4 were heavily localized in L4c and L5/6. Anatomical studies of L4c stellate cells suggest a strong axonal arborization toward L2/3 (74). Similarly, both dendritic fields and axon paths of many L5/6 neurons are found in supragranular and infragranular layers (16). We believe such morphologies are likely to show more propagation of activity away from the soma and may explain why we see most broad-spiking neurons have asymmetric multichannel waveforms.

### Limitations.

Some limitations of our approach should be noted. First, different anesthetics can have distinct effects on neural activity in-vivo. We used isoflurane while other studies used sufentanil (27, 45). Second, time constraints under anesthesia limited us to only grayscale stimulation, which precluded us from identifying functional neuron types that are involved in more visually complex pathways (e.g. layer 2/3 blobs which receive thalamic input from koniocellular neurons). Third, we took advantage of Neuropixels’ ability to provide extracellular waveforms across multiple channels. However, even the 20 μm interelectrode spacing may have missed very small neurons, such as VIP interneurons, and limited the full characterization of the multichannel profile. Fourth, our WaveMAP classification remains unvalidated, only providing candidate cell classes.

We believe these limitations will soon be overcome as experimental and analytical approaches evolve. Neuropixels are now widely used in the behaving monkey with a wide range of stimuli (75). New electrodes such as Neuropixels Ultra may enable more precise localization, cleaner signal-to-noise ratios, and better multichannel waveforms (76). Finally, multiple labs have developed AAV viral constructs with enhancers specific for inhibitory neurons (77–79), and projection targets in vivo (80). We recently used these constructs in anesthetized animals and reliably optotagged inhibitory neurons in the premotor and prefrontal cortex (81). Future experiments will be able to combine optotagging with our approaches here and better delineate the role of V1 cell types during visual behavior.

## Methods

Detailed methods are available in *SI Appendix*. *Methods* are adapted from ref. 28 as the same dataset is reanalyzed in this study but with a focus on candidate cell types and their properties. For completeness and readability, we replicate some of these methodological details, but the majority of the methods focuses on key details about the WaveMAP approach and functional assessments.

## Supplementary Material

Appendix 01 (PDF)

## Data Availability

Code to generate figures has been deposited in Github (https://github.com/carrn/V1_waveMAP) (82). Previously published data were used for this work (https://doi.org/10.5061/dryad.x3ffbg7p2) (83).
