# Supplementary material for: Neuropixels reveal laminar microcircuit organization in monkey V1 in vivo
Source: Proc Natl Acad Sci U S A. 2026 Feb 18;123(8):e2521556123. doi: 10.1073/pnas.2521556123 (PMC12933057; doi:10.1073/pnas.2521556123)
Supplement: Supplementary file 1 — Appendix 01 (PDF) [file pnas.2521556123.sapp.pdf]

PNAS

Supporting Information for

Neuropixels reveal laminar microcircuit organization in monkey V1 *in vivo*

Nicole Carr, Shude Zhu, Xiaomo Chen, Kenji Lee, Alec Perliss, Tirin Moore, and Chandramouli Chandrasekaran

Chandramouli Chandrasekaran.
E-mail: cchandr1@bu.edu, tirin@stanford.edu

This PDF file includes:

Supporting text
Figs. S1 to S6
Table S1
SI References

Supporting Information Text

Methods

Several methods sections are adapted from Trepka et al. (2022)(1) as the same dataset is re-analyzed in this study but with a focus on candidate cell types and their properties. For completeness and readability, we briefly replicate some of these methodological details here. The majority of the methods focuses on key details about the WaveMAP approach and functional assessments.

Subjects. The dataset used in this paper is composed of electrophysiological recordings in 2 anesthetized adult male rhesus macaques (Macaca Mulatta, M1, 13 kg; M2, 8 kg). Experimental data were collected under procedures that were designed in full accordance with National Institutes of Health Guide for the Care and Use of Laboratory Animals, the Society for Neuroscience Guidelines and Policies. All experimental and surgical procedures were approved by the Institutional Animal Care and Use Committee (IACUC) protocol (APLAC-9900) of Stanford University.

Surgical Details and Electrophysiological Recordings.

Surgical Details. Our workflow for the surgery and electrophysiological recordings were as follows. Twenty-four hours before the recording session, the monkeys were treated with dexamethasone phosphate to reduce cerebral edema. Then, on the day of the recordings, we sedated the animals using ketamine HCl (10 mg/kg body weight, intramuscularly). Monkeys were then intubated and ventilated with 1-2% isoflurane in a 1:1 mixture of nitrous oxide and oxygen to maintain general anesthesia, and placed in a stereotaxic frame. During the surgery, we monitored electrocardiogram, respiratory rate, body temperature, blood oxygenation, end-tidal CO_2, urine output and inspired/expired concentrations of anesthetic gases to ensure stable anesthesia. Normal saline was administered intravenously at a variable rate to maintain adequate urine output. Once animals were stable on the anesthetic plane, we administered a cycloplegic agent (1% atropine sulfate for pupil dilation) and focused the eyes with contact lenses on an LCD monitor. Finally, we used vecuronium bromide (60 μg/kg/hr) to prevent eye movements.

Electrophysiological Recordings. We first performed an occipital craniotomy over the opercular surface of V1 and then reflected the dura to expose a small (~ 3 mm^2) patch of cortex. Next, we identified a region relatively devoid of large surface vessels and inserted the Neuropixels probe with the aid of a surgical microscope. The Neuropixels 1.0 probe is quite thin (70 μm x 20 μm) and sometimes flexed upon contacting the pia. Thus, insertion sometimes required multiple attempts if it flexed upon contacting the pia. The junction of the probe tip and the pia could be visualized via the (Zeiss) surgical scope and the relaxation of pia dimpling was used to indicate penetration, after which the probe was lowered at least 3–4 mm. Prior to probe insertion, we dipped the Neuropixels probe in a solution of the DiI derivative (FM1-43FX, Molecular Probes, Inc.). The dye was used for subsequent fluorescent histological visualization of the electrode track (e.g., Fig. S1A-D).

The Neuropixels 1.0 probe has 986 contacts throughout the length of the probe (1 cm), of which 384 can be selected at any given point for recordings. We selected either the opercular surface cortex (M1) or within the underlying calcarine sulcus (M2). Recordings were made at 1–3 sites in one hemisphere of each monkey, ∼8-10 mm posterior to the lunate sulcus, and ∼5 mm from the longitudinal fissure. At the end of the experiment, monkeys were euthanized with an overdose of pentobarbital (150 mg/kg) and perfused with normal saline followed by 1 liter of 1% (wt/vol) paraformaldehyde in 0.1 M phosphate buffer, pH 7.4.

Visual stimulation. Visual stimuli were presented on a LCD monitor NEC-4010 (88.5 (H)* 49.7 (V) cm, 1360 × 768 pixels and a frame rate of 60 Hz) positioned 114 cm from the monkey. We first identified receptive fields (RFs) from online multi-unit activity and localized on the display using at least one eye. RF eccentricities were ∼4–6° (M1) and ∼6–10° (M2). We then presented circular drifting Gabor gratings (2°/sec., 100% Michelson contrast, 1.5 degrees of visual angle in diameter) within the joint RFs of recorded neurons either monocularly or binocularly. Gratings drifted in 36 different directions between 0–360° in 10° steps in a pseudorandom order. We used four spatial frequencies (SF, 0.5, 1, 2, 4 cycle/°). Optimal SFs and eye conditions were determined offline to categorize V1 neurons into simple or complex neurons. We used a stimulus duration of 1 second and repeated each stimulus 5 or 10 times and presented a blank screen with an equal luminance to the Gabor patch during the inter-stimulus interval (0.25 s).

Laminar Boundary Assignments.

Current Source Density with Histology. We estimated the depth of each unit relative to the boundary between L4c and L5, using the current source density analysis and histology results identifying the electrode tracks. This process is outlined in more detail in Trepka et al. (2022) (1), and we provide a brief description here. For each recording, we first performed the current source density (CSD) analysis on the stimulus-triggered average of LFP. LFP signals recorded from each of the 4 neighboring channels were averaged and realigned to the onset of the visual stimulus. CSD was estimated as the second-order derivatives of signals along the probe axis using the common five-point formula (2). The result was then smoothed across space ($\sigma = 120$ μm) to reduce the artifact caused by differences in electrode impedance. We located the lower boundary of the major sink (the reversal point of sink and source) as the border between L4c and L5. Based on this anchor point, we assign other laminar compartment borders using histological estimates by a neuro-ophthalmologist, Dr. Jonathon Horton (Fig. S1A-D). The border between L5 and L6 was calculated as the midpoint between the L4c/5 boundary and the L6/WM boundary. Our focus here was on relating functional properties of neuron populations to their laminar positions in the V1 microcircuit. Therefore, we did not

use a recently published method that uses the action potential band and unit densities (3) for laminar assignment in monkey V1 that uses LFP instead of CSD to assign layers because the logic would be circular.

Additionally, although in V1 layers 2, 3, 4a, 4b, 4cα, 4cβ, 5, and 6 are distinct layers, we do combine certain layers in order to have the neuron yield possible to make statements about populations of neurons with distinct functional activity in V1. Layers 2 and 3 were undersampled from M2, likely due to probe penetration depth, and needed to be combined. Layer 4a was very small in the area that we recorded from (Fig. S1A-D), and also needed to be combined. In Table S1, we show the number of units in each cluster that passed curation separated by layer (see *Spike Sorting and Data Curation* below). With 9 clusters and 6 layers, we unfortunately did not have sufficient power to separately analyze the neurons/clusters in layer 5 from those in layer 6. Therefore, for this study we combine layers 2 and 3, 4a and 4b, 4cα and 4cβ, and 5 and 6 for sufficient power during all of our laminar and functional analysis.

Scaling Laminar Depths. For the scaling of each session, the layer boundaries of all five sessions were averaged to obtain a common reference of laminar organization. For each session, the units within each layer assignment were scaled to their equivalent depths within the average boundary assignment, similar to previous layer normalization techniques (3, Fig. 1F). In sessions 4 and 5 (M2), the probe recorded from the calcarine sulcus, and all depths were inverted before scaling. It is important to note some experimental differences between sessions, including that sessions 4 and 5 did not have many units recorded from layers 2/3 (n = 5 units) due to penetration depth, and session 2 showed minor drift in its receptive field properties (4).

To ensure that all recordings across both animals were in V1, we automatically mapped RFs in session 4 from M2, and found that most of the RFs are around 0.4*0.4 dva in size, and during manual experiments, we were unable to find good RF locations for the other eye. These observations also suggested the M2 recordings more likely to be V1 than V2. When looking at a more zoomed out image of the session 5 insertion site with arrows registering corresponding blood vessels (Fig. S1D), we can see that our probe trace is in calcarine V1. Additionally, our functional analyses of the receptive field properties (see *Single Neuron Response Properties* below) suggests that all 5 recording sessions were within V1 and largely perpendicular to the surface of cortex. First, we find that the orientation tuning curves separated by monkey show similar tuning profiles (Fig. S1H). Second, in our previous analysis of these same sessions (1), we found a strong concentration of simple cells. Here again, both monkeys had near identical ratios of simple to complex indices (Fig. S3B). In addition, when we separate the simple/complex indices by cluster (Fig. S3D) by monkey, we see similar distributions. Third, for both monkeys, we observe faster responses in layer 4c than the other layers (Fig. S3A). These analyses show consistent results across both animals, supporting that both M1 and M2 recordings were from V1.

We did several statistical tests to ensure that the laminar distribution of our clusters is significant. We performed a chi-square test to determine if the observed number of positive spiking neurons neurons and negative spiking neurons in cortex and white matter were different from the null distribution (where all recorded neurons are equally distributed in cortex and white matter). We performed a single factor ANOVA test to determine if significant differences existed among all clusters for the scaled depths. We also performed a shuffle test to see if the number of narrow-spiking neurons (see *WaveMAP Analysis* below) observed in L4a/4b and L4c was significantly different from the number of narrow-spiking neurons in L4a/4b and 4c when depth was randomly assigned. In each shuffle, neurons with cluster assignments intact were randomly reassigned depths (1000 shuffles). In each shuffle, we maintained the cluster labels, but randomized the depths. We then counted the number of NS neurons in each of the laminar compartments and calculated a percentage. We did this 1000 times and found that the unshuffled percentage of NS neurons in layer 4a/4b and 4c was outside the range of the shuffled data and thus significant ($p < 0.002$, $2/n_{Shuffles}$) We performed a chi-square test to determine if the observed number of narrow-spiking neurons in L2/3, L4a/4b/4c, and L5/6 were different from the null distribution (where all neurons are equally distributed in cortex). Finally, we performed a chi-square test to determine if the observed number of neurons in each laminar compartment and each cluster (see *WaveMAP Analysis* below) were different from the null distribution (where all neuron classifications are equally distributed in all layers of cortex, Fig. S4B, D).

Spike Sorting and Data Curation. We used Kilosort2 to extract waveform templates of the extracellular waveform of each unit (5). We show the drift maps in all sessions (Fig. S1F, G). Sessions 2, 4, and 5 showed very stable recordings, presumably because they were recorded from deeper cortex (calcarine sulcus). Sessions 1 and 3 showed some slow drift, which was tracked by the Kilosort2 algorithm. Any fast, occasionally big jumps are more likely to be noise because of insufficient spikes to estimate drift. Those drifts are bigger than previously reported (6), but here we are using different probes (rodent probe with 70μm vs. NHP of 125μm in width of shank). We also did not use any stabilization procedures (blunt guide tube, etc). We also report the ISI violations per session (Fig. S1E). After Kilosort2, we isolated the maximum amplitude channel from the template for each unit, as well as 10 channels above and 10 below the maximum amplitude channel for the multichannel trajectory (*see Multichannel Profiles below*). Each maximum amplitude waveform was normalized between 1 and -1. Finally, we separated the dataset into positive and negative spiking units, with the maximum peak of positive units occurring before the minimum trough.

To convert Kilosort2 template projection amplitudes to a good estimation of raw amplitude, a scaling factor from the acquisition system of 2.3 was applied for units of μV. For additional spike sorting and the WaveMAP analysis, we used the normalized waveform template to emphasize local features of the extracellular action potential shape, rather than be biased by amplitude differences among all neurons. However for any analysis relating functional responses to the amplitude of the neuron, we used the raw (non-normalized) amplitude.

In our past experience, we have found that rigorous and conservative spike sorting is a prerequisite for delineating cell

types using WaveMAP (7). To obtain high-quality single units that could then be passed to WaveMAP, we used the following semi-automatic quality control method (Fig. 1B). This method involved two parallel steps. In the parallel step 1, we developed a graphical user interface (GUI) to manually curate all the units. The GUI presented the normalized maximum amplitude waveform, as well as three user selectable annotations: "good", "noisy", and "artifact". For this step, two reviewers (NC and AP) curated all 2,529 templates from all 5 sessions in the first pass. The waveforms that were rated "good" by both reviewers were confirmed, and then any units that received a single "good" label or "noisy" by either reviewer were run through a second pass of inspection. Finally, we only chose units that were annotated as "good" by both reviewers. We calculated both internal consistency through Chronbach's Alpha ($\alpha = 0.72$), and the inter-rater reliability through Cohen's Kappa ($\kappa = 0.69$), which gave us acceptable agreement on internal consistency, as well as substantial agreement for inter-rater reliability. In the parallel step 2, we filtered units by SNR and excluded units with SNR thresholds below 0 or above 3.7 (95% CI: [0.3350 1.0641]). The final dataset was the intersection of the units that passed both the manual curation step and the SNR threshold. After this quality control, our final dataset (regardless of visual responses) with positive and negative spiking waveforms was composed of 905 units (Fig. 1C).

WaveMAP Analysis. We applied our novel WaveMAP approach (7, 8) to our dataset, with positive and negative units separated to prioritize local over global clustering. We first applied UMAP to the normalized maximum amplitude waveforms after spike sorting and quality control to obtain a graph, and then passed this graph to Louvain clustering to delineate clusters (N_neighbors = 20; MIN_DIST = 0.2; RESOLUTION = 1.0). More technical details of the WaveMAP approach are available in the original publication (8). The 801 negative spiking units were partitioned into 9 clusters. NS-1, NS-2, NS-3, and NS-4 were narrow-spiking, TP-1 was tri-phasic, and BS-1, BS-2, BS-3, and BS-4 were broad spiking. The 104 positive spiking units were partitioned into 5 clusters (Fig. S6A).

We used a five-fold cross-validation classification approach to assess whether the identified clusters were well separated from each other, and found that it was indeed the case (88% standard accuracy over 9 clusters), which suggests we did not overcluster on this dataset (Fig. 2C).In addition, the Louvain clustering approach is a hierarchical approach, and so higher resolution parameters lead to increased importance on global vs. local features, and larger and fewer clusters. Thus, when applying a higher UMAP resolution parameter (RESOLUTION = 2.5), our narrow-spiking neurons combined into one large cluster showing that the putative cell classes in the narrow-spiking group are closer to each other in high-dimensional space than to the other groups (Fig. 2D).

We also used an alternative clustering approach termed ensemble clustering for graphs (ECG, 9)), a consensus clustering method that simultaneously evaluates various resolution parameters, and we found that this clustering also resulted in nine candidate cell types and largely overlapped with our UMAP approach (Fig. 2E). We calculated a MARI index between the ECG and the 1.0 resolution WaveMAP clustering, to give a measure of how well the methods overlap. MARI is a function which computes the modified adjusted Rand index of two classifications, with a value of 1 for identical clustering, and a centering around 0 for random assignments (10). This method is well suited for fuzzy unsupervised clustering, with unknown cluster sizes. Indices of 0.7 to 0.8 are considered excellent in terms of cluster overlap, with high stability (8). Thus, the 9 clusters found using Louvain clustering with a resolution of 1.0 and the ECG have reasonable similarity (although not identical).

To better understand why WaveMAP separated these waveforms into these clusters, we performed three classical operations on the waveforms of this dataset. First, we calculated classical features of the waveform, including the trough-to-peak width, or the time between trough and peak, the trough-to-peak amplitude, which is the raw voltage change between the trough and the peak, and the repolarization time, defined as the time between the peak and 1/2 the peak of the repolarization curve (Fig. S2A). We then scaled the size of each unit point in UMAP space by the width of the waveform, the time for repolarization, and the non-normalized amplitude (Fig. S2B-G). This plot revealed that both UMAP dimension 1 and 2 were positively correlated with both waveform duration and repolarization time using Pearson linear correlation. An important step in pre-processing waveforms for WaveMAP is to normalize the waveforms so amplitude should not factor in the clustering. Nevertheless, when we scale the size of the markers of each unit by its raw, or non-normalized amplitude, we found that WaveMAP identifies that some clusters have larger amplitudes than others (Fig. S2F), again highlighting that our approach can separate clusters with biologically relevant differences.

Single Neuron Response Properties. To characterize neuronal properties, the evoked activity was assessed using mean firing rate (spikes/sec) over the whole stimulus presentation period, offset by response latency delay. Only responses to the preferred spatial frequency and eye conditions were selected. The maximum firing rate was the neuron's response to the preferred drifting orientation and direction.

Receptive Field Properties. We assessed the following receptive field properties. These properties have been used extensively in past literature to characterize V1 neurons *in vivo* and provide a robust assessment of functional properties.

1. Direction selectivity (Direction Index, DI) was determined as the response to preferred orientation and drift direction minus the response to preferred orientation but opposite drift direction, divided by the sum of these two responses (11).

$$DI = \frac{FR_{prefdirection} - FR_{nulldirection}}{FR_{prefdirection} + FR_{nulldirection}} \quad [1]$$

2. Orientation selectivity (Orientation Index, OI) was determined as the response to preferred orientation minus the response to orthogonal orientation, divided by the sum of these two responses (11).

$$OI = \frac{FR_{preforientation} - FR_{nullorientation}}{FR_{preforientation} + FR_{nullorientation}} \quad [2]$$

3. Simple Complex Index, also referred to as Modulation Ratio in prior studies, was defined as F1/F0, where F1 and F0 are the amplitude of the first harmonic at the temporal frequency of drifting grating and constant component of the Fourier spectrum to the neuron's response to preferred orientation. A simple-complex index of less than 1 was assigned as complex cell, and greater than 1 as a simple cell (12, 13).

We performed single factor ANOVA tests to determine if significant differences existed among all clusters for functional properties and the bursting index (see *Bursting Index* below). We also performed a shuffle test to see if the median values for amplitude and direction index for each cluster/layer population were significantly different from the shuffled layer and shuffled cluster data. In each shuffle (1000 shuffles), neurons were randomly reassigned clusters and layer compartments to create a null distribution, which we compared the median amplitudes and direction index of each cluster/layer compartment against. Finally, we performed PCA on functional properties including orientation index, orientation circular variance, direction index, direction circular variance, orientation tuning bandwidth, and simple complex index. We then used multiple linear regression to see what factors explained the variance in this data (Fig. S2H).

Peri-stimulus time histogram - PSTH. For each unit, we calculated a peri-stimulus time histogram (PSTH) for all conditions between the period of 50 ms before and 500 ms after stimulation. 67 ms were added to the stimulation time to correct for the time for the screen to update the visual stimulus, and we Gaussian smoothed with a 13 ms kernel. The stimuli presented were session dependent, with either 144 conditions (monocular stimulation for sessions 1, 4) or 432 conditions (monocular and binocular stimulation for session 2, 3, 5). There were 5-10 repeated stimuli per session. A trial-averaged "preferred stimulus" PSTH was estimated for each unit in spikes/s. This was determined by selecting the condition with the highest firing rate in the stimulus period, then all trials of that condition were averaged together for that unit's "optimal" PSTH.

For each cluster, an average PSTH included the "preferred stimulus" PSTH of each unit in that cluster (Fig. S3C). For each layer, an average of the "preferred stimulus" PSTHs of all units within the bounds of the layer was calculated (Fig. S3D). The standard error of the mean was calculated and reported in both the layer and cluster PSTH analysis.

Inter-spike Interval. We estimated the burstiness of the units by analyzing the inter-spike interval (ISI) distribution. The inter-spike interval was estimated using the difference in all spike times per unit throughout the entire recording session, with 0.4 ms bins. We chose this bin size as it most clearly represented the structure of the ISI density distributions, without overemphasizing random noise or artifacts. The ISI histograms were normalized to a relative probability, where the number of elements in each bin relative to the total number of elements in that bin is 1 at maximum. Visual inspection suggested considerable heterogeneity in these ISI distributions (Fig. 5B).

Bursting Index. To analyze this heterogeneity, we used principal component analysis (PCA) to determine the various components of these ISI distributions (Fig. 5C). We then estimated the principal component coefficients for each unit. We plot the mean loadings of each cluster and their standard error of the top three principal components against each other (Fig. 5D). The difference between the first and third PC loadings make up our bursting index, which we plot per unit against laminar depth and orientation selectivity. Finally, we plot the median orientation index and bursting index per cluster against each other, and performed a shuffle test (1000 shuffles) for both indices to determine any clusters with median orientation index or median bursting index outside of the 1st and 99th percentiles of the shuffled distribution (Fig. 5E, F).

Multichannel Profiles. The extracellular action potential waveform template from 10 channels above and 10 channels below the maximum amplitude channel were extracted to build the multichannel profile per unit. Due to the channel mapping (checkerboard pattern), a unit was located on either the left or right side of the probe (Fig. 1A). Only the column of channels to which the maximum amplitude unit waveform belonged (left or right side of probe) was included in calculations of propagation trajectory, leaving 5 channels above and 5 channels below (Fig. 6A). The trough trajectory was the time of the minimum value on each channel as a function of the channel depth relative to the maximum amplitude channel, only including data within the 5th and 95th percentile (Fig. 6C). We use a trim mean for the trough-time trajectory per cluster (by only including data within the 5th and 95th percentile to avoid outliers) with SEM errorbars. There were a number of units on channels with no signal, or were on the edges of the probe (lacking 5 channels above or below the center channel), creating outliers in the multichannel data. By excluding the outliers when calculating the mean, we ensure that cells that have extremely small waveforms do not lead to arbitrarily long trough times as they are further away from the maximum amplitude channels. The best fit slope of the mean trough trajectories represented the trough propagation velocity (mm/ms) of each cluster (see *Propagation Trajectories and Velocities* below). Qualitative assessments of the trough propagation velocities above and below the soma may indicate morphological features of the neuron, such as alignment of the neuron's apical dendrite to the probe or how symmetric the velocities are.

Propagation Trajectories and Velocities. A time-domain analysis of the troughs of the multichannel profile shows mean propagation trajectories of each cluster (only including the 95th percentile of all trough times to exclude outliers), with errorbars showing standard error of the mean trough time at each channel per cluster. We used linear regression to calculate the slope of the mean propagation trajectories above and below the soma for each cluster, which are referred to as propagation velocities (14, Fig. 6D). The propagation velocity is reported in units of (ms/mm), and so is also referred to the inverse velocity (velocity^{-1}). We used bootstrapped trough times (500 resamples) for each cluster to find the standard deviation of the propagation velocities per cluster, or the bootstrap SE. For each cluster, we plotted the mean propagation velocity above and below the soma against each other (velocity$^{-1}{}_{below}$, velocity$^{-1}{}_{above}$) to gain a metric of velocity symmetry around the soma. On the velocity$^{-1}{}_{below}$ axis, if a cluster is located higher than 0, the slope below the soma continues in the same direction as above the soma, meaning the propagation velocity is unidirectional. If it is less than 0, the velocity slope below the soma is in the opposing direction than the slope above the soma, meaning the propagation velocity is bidirectional. We tested which clusters showed a significant difference in propagation velocity below the soma from 0 using independent bootstrap tests with 98% confidence intervals and 500 resamples (Fig. 6F). On the velocity$^{-1}{}_{above}$ axis, the slope increases roughly in the order of peak-trough duration.

Asymmetry Index. We calculated the index of symmetry (SI) as the orthogonal distance between each point (velocity$^{-1}{}_{below}$, velocity$^{-1}{}_{above}$) and the diagonal line y = -x (15). The equation is as follows:

$$SI = \frac{ax_0 + by_0 + c}{\sqrt{a^2 + b^2}} \qquad [3]$$

where (x_0,y_0) = (velocity$^{-1}{}_{below}$, velocity$^{-1}{}_{above}$), a = 1, b = 1, and c = 0 for y = -x. An unsigned asymmetry index was then calculated, which does not include information on which direction (above vs. below the soma) was dominant. A small value of the asymmetry index would indicate more symmetry between the velocity slopes above and below the soma, whereas a larger value indicates more asymmetry. A negative value indicates that the velocity slope below the soma is greater than the slope above the soma. We performed an independent bootstrap test (500 resamples) for significant differences between all clusters, plotted in a heatmap that indicates a significant difference between pairs with a 95% confidence interval (Fig. 6E).

We performed an additional analysis to recreate the asymmetry index analysis so that instead of 5 channels above and below, we look at 2 channels above and below the center channel (Fig. S5D-G). If our results were just a result of differences in waveform amplitude further from the maximum amplitude channel then the effect should not be replicated by just using 2 channels above and below the maximum amplitude channel. We found that even in this case the clusters that we highlighted as exhibiting asymmetric and unidirectional propagation are still strongly asymmetric and unidirectional (NS-1, BS-1, BS-2, and BS-4). The TP-1 cluster still retains its large variance and symmetric propagation velocities (perhaps due to the different types of TP cells we encountered). Finally, the clusters we believe to have more stellate-like properties (NS-3 and NS-4) are still symmetric, albeit with modest changes. NS-3 is slightly less symmetric and NS-4 is slightly more symmetric (Fig. S5D-G). Note, some of these issues may be even better resolved with the Neuropixels Ultra, which has 10-fold higher spatial resolution, which a recent publication has used in mouse V1 (16).

Cross-Correlations and Connectivity. Cross-correlations between spike trains of all pairs of simultaneously recorded neurons were computed (1). Spiking activity was chosen within the 0.4–1 s window of each visual stimulus presentation. The raw cross-correlogram (CCG) for a pair of neurons (j,k)was defined as follows:

$$CCG(\tau)_{j \leftarrow k} = \frac{\frac{1}{M}\sum_{i}^{M}\sum_{t=1}^{N-\tau} x_j^i(t) \times x_k^j(t+\tau)}{\theta\tau\sqrt{\lambda_j \lambda_k}} \qquad [4]$$

where M is the number of trials, N is the number of time bins within a trial, τ is the time lag, $x_{ij}(t)$ is one if neuron j fired in time bin t of trial i and zero otherwise, and λ_j is the mean firing rate of neuron j computed over the same bins used to compute the CCG at each time lag. $\theta(\tau)$ is a triangular function, $\theta(\tau) = N - |\tau|$, that corrects for the difference in the number of overlapping bins at different time lags. We denote the CCG computed with neuron j as the first (reference) neuron and k as the second (target) neuron in the correlation function as j-k.

Raw cross-correlograms (CCGs) were normalized by the geometric mean of firing rates of the pairs and then jitter-corrected to remove the fluctuations slower than 25ms. For excitatory interactions, a CCG was determined significant if its peak occurred within 10 ms of zero-time lag, and its peak value exceeded 7 standard deviations above the mean of the noise distribution (defined as CCG values of 50-100 ms from zero-time lag).

Lead-Lag Index. The lead-lag index is a measure of correlogram asymmetry (CA), calculated as the values on the right (lead) side of the CCG, subtracted by the left (lag) side, and then normalized by their sum (e.g., a value closer to 1 means that the reference cell is leading the target cell). If the CA is within [-0.3, 0.3], the peak lag is $\leq$ 1 ms, or the peak width is $>$ 4 ms, the CCG is more symmetrical, reflecting a common input, whereas a CA larger than 0.4 or smaller than -0.4 may reflect synaptic connections (17). We show histogram and cumulative distributions of peak lags and peak widths with thresholds to show that the majority of significant CCGs reflect feedforward interactions rather than purely common input. We report the median peak lag and peak width for neurons with lead-lag index (CA) $\leq$ 0.3 and lead-lag index (CA) $>$ 0.3. We then test if these two groups have significantly different peak lag, and peak width with a Wilcoxon rank sum test. Overall, these results suggest

that the majority of CCGs likely reflect cell-to-cell interactions, and that the lead-lag connections between clusters remain significant and reflect feedforward trends.

The lead-lag index was calculated for each CCG between two units from different clusters. To get a measure of lead-lag directionality between clusters, the median lead-lag index was calculated for each cluster pair (Figure 7). For each cluster pair or cluster/layer pair, the distribution of significant CCGs between two clusters were plotted. We then calculated the median, and did a Wilcoxon rank sum test to test whether the median is different from 0.

Supplementary Discussion

Potential for *in vivo* identification of PV population. We identified four narrow-spiking clusters with distinct properties. Do any of the clusters map onto a parvalbumin (PV) specific inhibitory population? Anatomical studies of V1 suggest that PV neurons are observed in all layers, but are more likely in L4a, L4b, and L4c. Currently, there are no functional studies from monkey V1 of PV neurons with ground truth optotagging. However, *in vivo* mouse studies suggest that PV neurons in V1 have narrow, small amplitude waveforms with symmetric forward and back-propagation profiles (15). Based on these observations, we suggest NS-4 (and likely the small amplitude neurons in cluster NS-3 not located in L4a/4b) are likely to be PV neurons. This assertion is based on five lines of evidence. First, NS-4 neurons were found throughout all the layers of cortex. Second, they also were one of the narrowest clusters observed with a mean trough to peak width of 0.24 ms. Third, they had symmetric multichannel waveforms as expected of aspiny cells with dense dendritic and axonal morphologies around the soma (14, 18). Fourth, they responded early after stimulus onset and had robust firing rates to stimulus onset that was earlier than all the broad-spiking neurons (19). Again, rigorous optotagging techniques (20) with inhibitory neuron specific viral constructs (e.g., those with mDlx 2.0 or other enhancers (21)) combined with Neuropixels recordings in monkey V1 will help establish whether PV neurons show the same layer distribution, and waveform properties as NS-4.

Tri-phasic waveforms and positive spikes likely emerge from return currents and axons. Our study thus far has largely focused on the interesting links between functional and structural properties of the biphasic negative waveforms (NS and BS groups). Our WaveMAP analysis also revealed extremely narrow tri-phasic waveforms (TP-1, with trough-peak duration = $\sim$170 μs) with very average functional responses, across the board. These waveforms were found both in gray matter in L4a through 6 as well as in white matter (Fig. S4B, D). For this TP-1 class, direction or orientation selectivity was largely uniformly distributed and unremarkable.

To better understand this TP-1 neural population, we examined the multichannel waveform of the TP-1 type and found two main types of profiles. The first, TP type A, had a localized multichannel waveform profile (Fig. S5A) and was present throughout cortex and white matter and was largely uniformly selective for orientation and direction. These neurons with a small and local multichannel waveform are likely balancing return currents when a spike is generated in the axon initial segment (22). The second type, TP type B, shows a much larger spread across channels (Fig. S5A), and these were mainly located within L4a/4b, L4c and L5/6 of cortex. They also had lower direction and orientation selectivity (Fig. S5B). The larger spread exhibited by TP Type B is reminiscent of recordings with Neuropixels along a retinal ganglion cell axon in the superior colliculus of the mouse (23). Thus, the multichannel subtype B of the TP-1 group is consistent with propagation through a neurite aligned vertically, or close to the axon initial segment.

We also found that positive spiking waveforms were quite common in the Neuropixels recordings. The majority of these positive spiking units were found in the white matter, with a smaller fraction in L5/6 (84%, χ^2 (1, 95) = 41.779, $p < 0.0001$, Fig. 1F). WaveMAP clustering on these waveforms revealed five clusters (Fig. S6A), all concentrated in white matter and deep L5/6 (Fig. S6B). We investigated the multichannel profiles of these neurons and observed a large spread across channels similar to TP type B (Fig. S6C). Functionally, these positive spiking neurons were largely unresponsive to visual stimulus (only 32/105 responsive units) and did not demonstrate any remarkable responses to the visual stimuli (Fig. S6D, E). Collectively, these results suggest that these positive spiking waveforms are likely axons passing through the white matter (3, 22, 24).

Supplemental materials

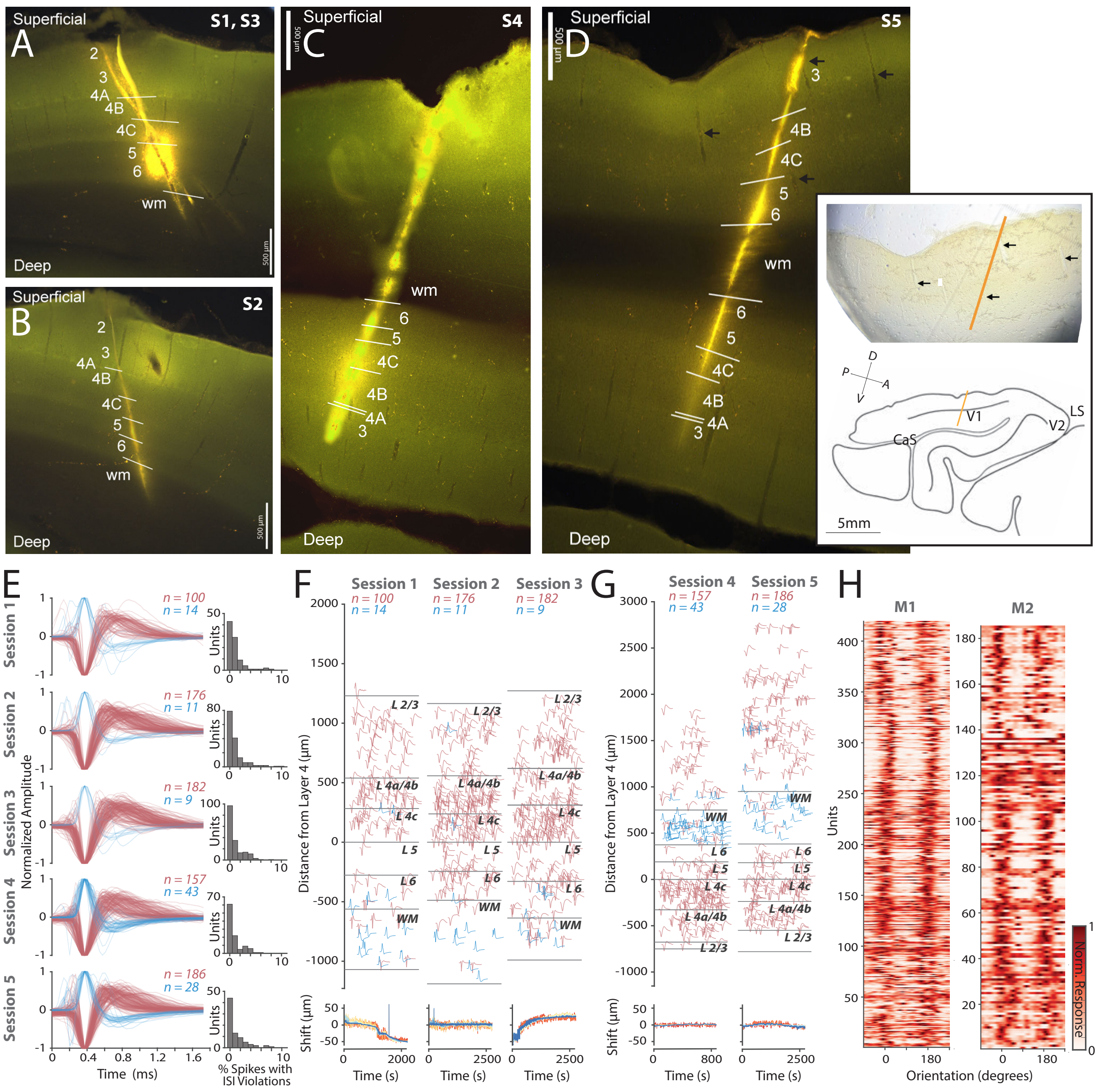

Fig. S1. Histological verification of penetrations and neuronal waveforms from each session (**A-D**) Dye tracks of monkey 1 (M1) for sessions 1 and 3 (**A**), session 2 (**B**). Dye tracks of monkey 2 (M2) for session 4 (**C**) and session 5 (**D**), all with delineations of laminar boundaries. The *top inset* in **D** shows zoomed out images of the insertion site with black arrows registering corresponding blood vessels. *Bottom inset*, Using the dip in the superficial tissue and the blood vessels as aligning features, the recording track can be seen (in orange) in the sagittal section of the M2 session 5 recording site. This recording site (session 5) was ∼10 mm posterior to lunate, and ∼5 mm to longitudinal fissure. The recording site for session 4 was from the left hemisphere of M2, ∼8 mm posterior to lunate, and ∼5 mm to longitudinal fissure. (**E**) *Left*, Curated waveforms for each of the five sessions. *Right*, Distribution of ISI violation rates (ratio of spikes with ISI $<$ 1ms out of all spikes, calculated per unit per session). Most units in each session show no ISI violations (0% ISI $<$1 ms) across the session. (**F-G**) Laminar organization of waveforms in sessions 1-3 (F, M1), and waveforms in sessions 4-5 (G, M2). The *bottom inset* shows the amount of probe drift throughout the session. (**H**) The normalized response tuning aligned to overall preferred orientation for 606 visually active neurons, sorted top to bottom by depth from superficial to deep, separated by monkey (M1 and M2).

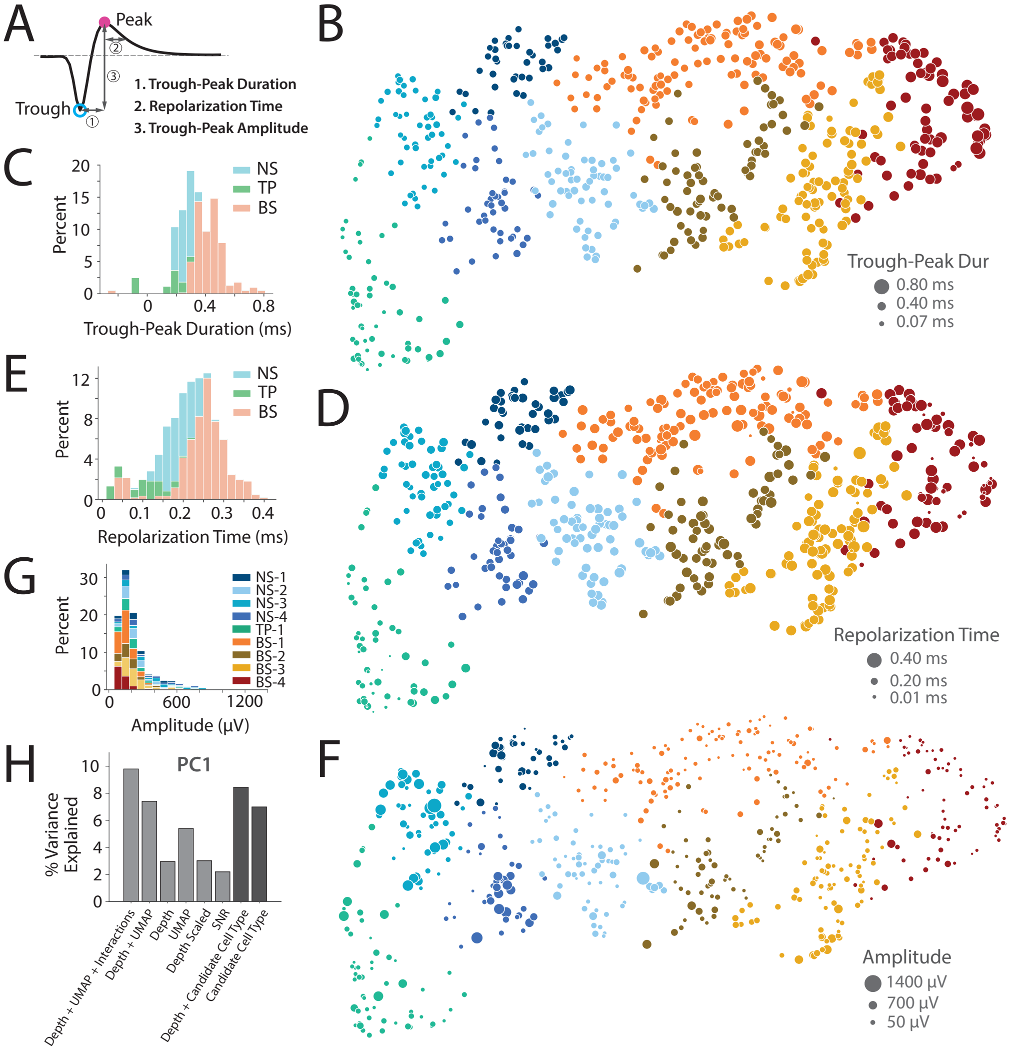

Fig. S2. WaveMAP clusters lawfully reflect classic features (**A**) Classic features of the extracellular waveform include peak (in pink solid circle), trough (in blue open circle), trough-peak duration (T-P Dur), repolarization time from 1/2 peak (Rep Time), and normalized trough-peak amplitude (T-P Amp). (**B**) UMAP X and UMAP Y plot with unit markers sized by trough-peak duration. UMAP X and Y are positively correlated with T-P Dur (Pearson's $r = 0.83$, $p < 0.0001$). (**C**) Histogram of all Trough-Peak Durations showing distributions of general categories (Tri-phasic, Narrow, and Broad). Note the distribution is unimodal and not easily separated into clusters. (**D**) UMAP X and UMAP Y plot with unit markers sized by 1/2 peak repolarization time. Again note the positive correlation of UMAP X and Y with repolarization time (Pearson's $r = 0.53$, $p < 0.0001$). (**E**) Histogram of all 1/2 peak repolarization times showing distributions of general categories (Tri-phasic, Narrow, and Broad). Note again the unimodal distribution of these features. (**F**) UMAP X and UMAP Y plot with unit markers sized by non-normalized trough-peak amplitude (Pearson's $r = -0.33$, $p < 0.0001$). The results suggest that some clusters have larger amplitudes than others, such as NS-3 and NS-4. (**G**) Histogram of all non-normalized amplitudes showing distributions for all clusters. (**H**) We performed PCA on the following functional properties of all visually responsive neurons to summarize the visual functional response of the neurons: orientation index, orientation circular variance, direction index, direction circular variance, orientation tuning bandwidth, and simple complex index. We examined if this functional visual response could be explained by various predictors of cell type using a multiple linear regression. The predictors we used are UMAP coordinates, Depth, and interaction terms between UMAP X coordinate and depth, UMAP Y coordinate and depth, and UMAP X, Y, and depth. As a control, we measured the amount of variance explained by signal-to-noise ratio (SNR) to ensure our results were not a trivial artifact of spike sorting. Overall, both UMAP X and Y, Depth and candidate cell type predicted 8.4% of the variance in visual functional responses. Notably, the candidate cell type label and depth explained comparable amounts of variance as the depth and the UMAP coordinates (9.8%).

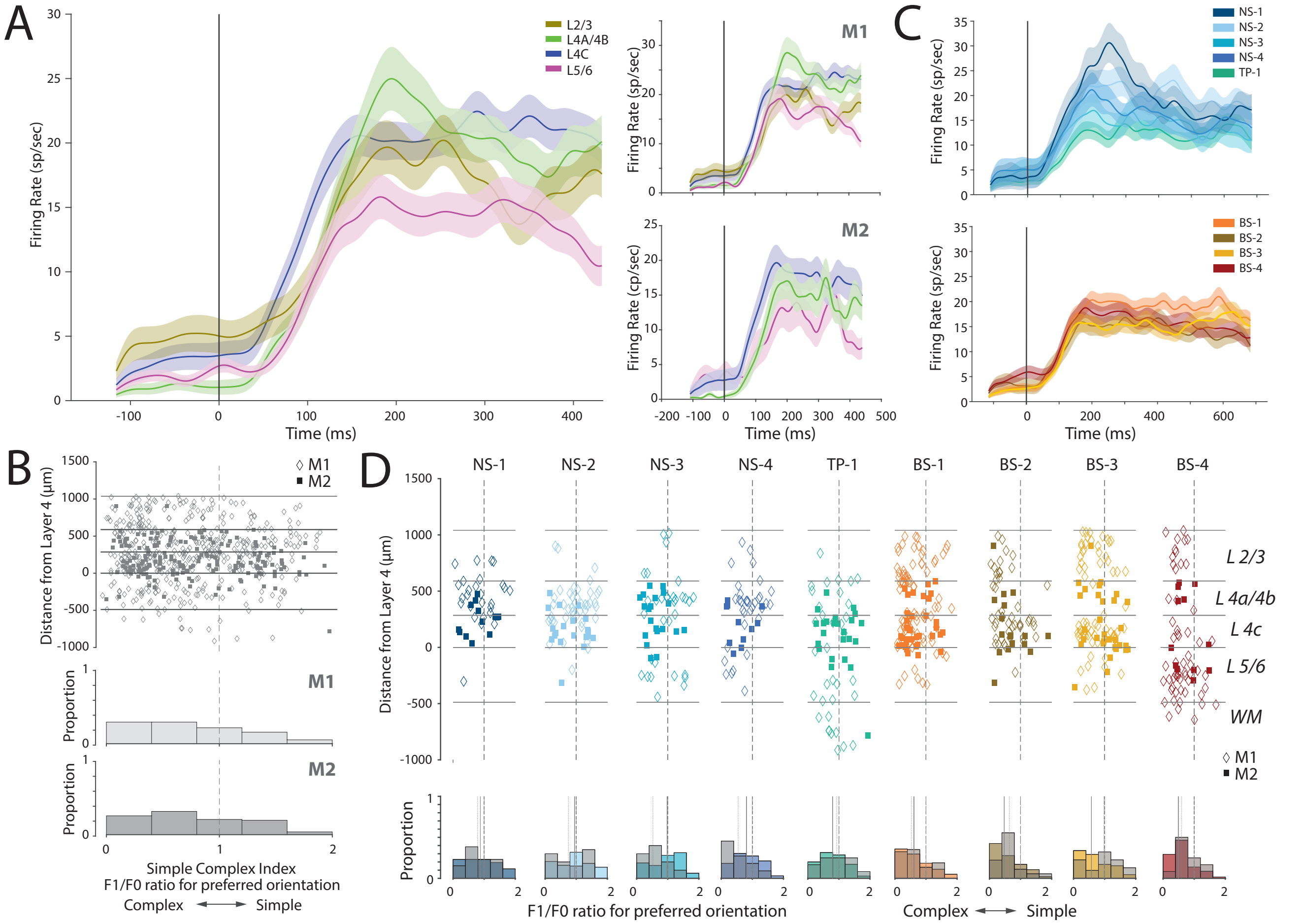

Fig. S3. Neuropixels recordings in V1 are consistent with classical studies (**A**) Average PSTH by layer. The black line indicates the initiation of the visual stimulus. *Top Inset*, Average PSTH by layer for sessions 1-3 from monkey 1. *Bottom Inset*, Average PSTH by layer for sessions 4-5 from monkey 2. (**B**) Scatter plot of modularity index (simple-complex index) as a function of scaled laminar depth of all units. Open diamond markers indicate sessions 1-3 from monkey 1, closed square markers indicate sessions 4-5 from monkey 2. Proportions by monkey show all sessions share the same distribution of simple and complex cells. (**C**) Average PSTH by cluster. The black line indicates the initiation of the visual stimulus. *Top*, Mean PSTHs of "narrow" clusters. *Bottom*, Mean PSTHs of "broad" clusters. (**D**) There showed no strong indications that simple and complex cells mapped onto any one candidate cell type, although the broad-spiking clusters generally trended towards more complex responses. *Top*, Scatter plot of modularity index (simple-complex index) and scaled laminar depth separated by cluster. Open diamond markers indicate sessions 1-3 from monkey 1, closed square markers indicate sessions 4-5 from monkey 2. *Bottom*, Panel below the scatter plots shows histograms of the simple-complex index. The solid color indicates sessions 1-3 from monkey 1 and the gray indicates sessions 4-5 from monkey 2. The vertical dashed center lines show the boundary between complex (left of center) and simple (right of center).

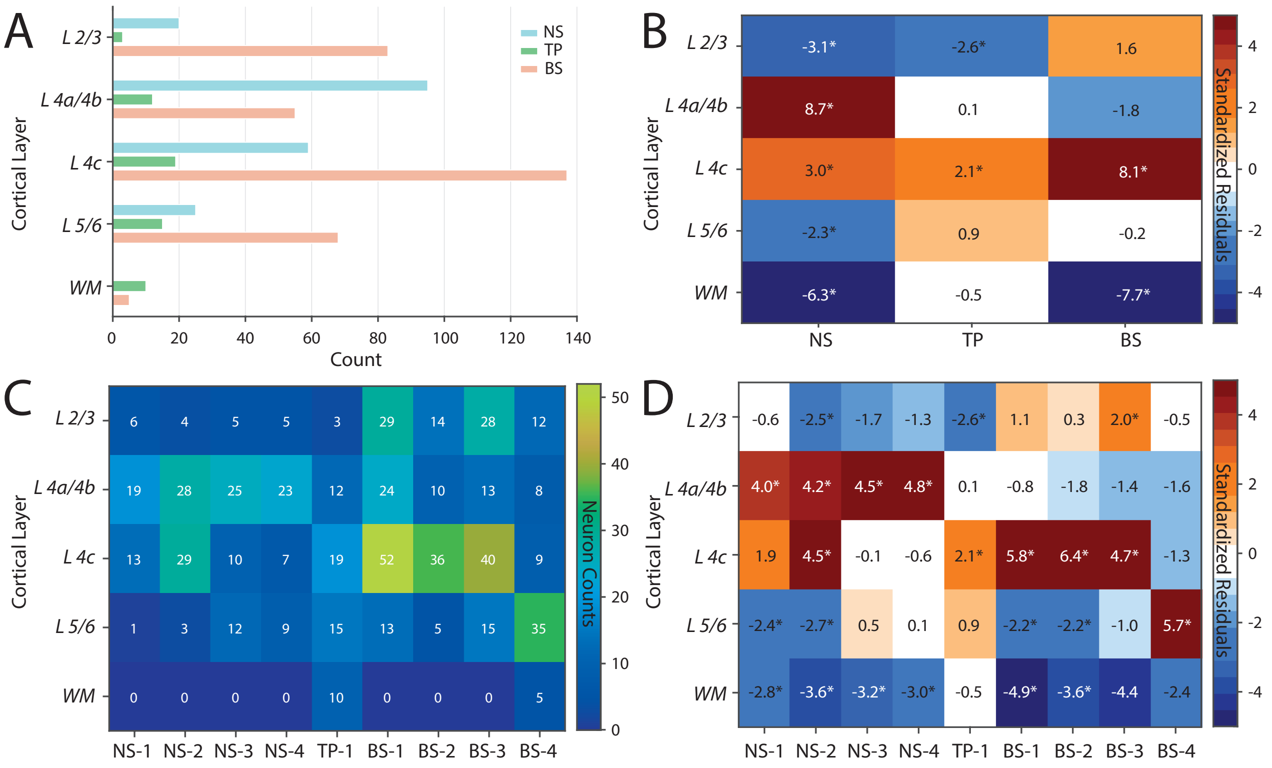

Fig. S4. Narrow-spiking groups are concentrated in Layer 4a/4b/4c (**A**) Histogram showing neuron count per layer separated by broad categories, narrow-spiking (NS), tri-phasic (TP), and broad-spiking (BS). (**B**) Under the null hypothesis that each layer (df = 4) should have the same proportion of neurons across the three broad categories (NS, TP, and BS), we performed a chi-square analysis to compare the observed distributions (NS Group: χ^2 (4, 199) = 140.74, $p < 0.0001$, TP Group: χ^2 (4, 59) = 12.11, $p = 0.016$, and BS Group: χ^2 (4, 348) = 131.29, $p < 0.0001$). The heatmap shows standardized residuals, which are the difference between the observed and expected, divided by the square root of the expected count. * indicates a value $\pm$ 2.0 which suggests significance at $p < 0.05$. A positive value indicates a greater number observed than expected, and a negative indicates a lower number observed than expected. We see that the NS category is overrepresented in L4a/4b and L4c. (**C**) Raw counts of neuron distribution by layer and cluster. (**D**) Under the null hypothesis that each layer (df = 4) should have the same proportion of neurons across each cluster, we performed a chi-square analysis to compare the observed distributions (NS-1: χ^2 (4, 39) = 33.70, $p < 0.0001$, NS-2: χ^2 (4, 64) = 64.97, $p < 0.0001$, NS-3: χ^2 (4, 52) = 34.01, $p < 0.0001$, NS-4: χ^2 (4, 44) = 33.82, $p < 0.0001$, TP-1: χ^2 (4, 59) = 12.11, $p = 0.016$, BS-1: χ^2 (4, 118) = 63.71, $p < 0.0001$, BS-2: χ^2 (4, 65) = 59.38, $p < 0.0001$, BS-3: χ^2 (4, 96) = 48.56, $p < 0.0001$, and BS-4: χ^2 (4, 69) = 42.47, $p < 0.0001$). The heatmap shows standardized residuals, which are the difference between the observed and expected, divided by the square root of the expected count. * indicates a value $\pm$ 2.0 which suggests significance at $p < 0.05$. A positive value indicates a greater number observed than expected, and a negative indicates a lower number observed than expected. We see that all NS subtypes are overrepresented in L4a/4b and NS-2, BS-1, BS-2, and BS-3 are overrepresented in L4c. BS-4 is overrepresented in L5/6.

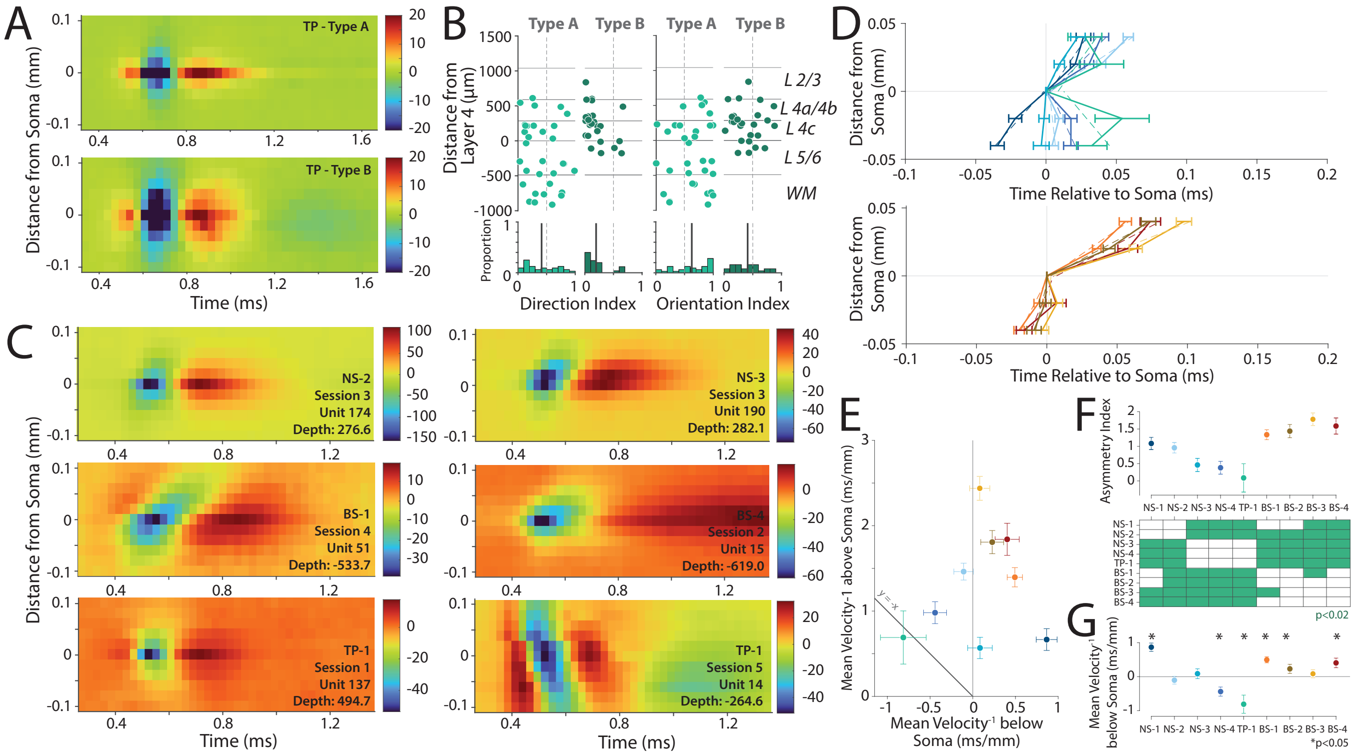

Fig. S5. Spatial profiles show more hetereogeneity than cluster features typically capture (**A**) Average multichannel waveforms of tri-phasic cluster Type A (n = 32), and Type B (n = 25). Type A and Type B were manually classified on the criteria of spread (number of channels on which an extracellular action potential waveform continuum was observed). Of the 59 TP-1 Waveforms that were visually responsive, 2 were identified as artifactual. (**B**) *Top*, Laminar distribution of TP-1 Type A and Type B. The x-axis shows (from left to right) direction index and orientation index. The Type B population has significantly less direction selectivity (Wilcoxon rank sum test, $p < 0.01$) and orientation selectivity (Wilcoxon rank sum test, $p < 0.05$) than Type A. *Bottom*, Panel below the scatter plots shows histograms of the direction and orientation selectivity index. The bold line indicates the median orientation and direction index for each cluster. The vertical dashed center lines show the boundary between not selective (left of center) and selective (right of center). (**C**) Example multichannel waveforms from various clusters and sessions. *Upper Left*, "Narrow" Cluster 2 waveform. *Lower Left*, "Narrow" Cluster 6 waveform showing backpropagation. *Upper Right*, Tri-phasic Cluster 7 waveform. *Lower Right*, "Broad" waveform showing backpropagation. (**D**) This is Fig. 6C-F redone with a smaller channel spread, since neurons with smaller spreads may naturally become less precise with increasing distance from the maximum amplitude channel. The trough trajectory, or the mean time of waveform trough at each channel location above and below soma (aligned to y-axis = 0, removing outliers using 95% confidence intervals) ± SEM. Velocities above and below soma and are estimated separately by linear regression (slope of the dashed line). *Top*, Narrow and Tri-phasic clusters. *Bottom*, Broad clusters. (**D**) Mean trough propagation velocity above soma vs below soma for each cluster. Clusters with positive slopes both above and below the soma are located on the right of x-axis = 0. Clusters which show positive slopes above and negative below the soma are located on the left of x-axis = 0. Errorbars are std calculated from bootstrapped data (500 resamples). (**E**) *Top*, Asymmetry index of each cluster (see *Methods Section: Asymmetry Index*). This is the absolute value of the distance of each cluster's propagation velocity above and below the soma, from the y=-x line. *Bottom*, Errorbars are bootstrap standard error (500 resamples). Independent bootstrap tests were done to compare the asymmetry index of each cluster, shown in a heatmap indicating significance (98% bootstrap CI, $p < 0.02$). (**F**) The mean slope of the propagation velocity below the soma was plotted per cluster. Errorbars are bootstrap standard error (500 resamples). Independent bootstrap tests were done to compare each slope to 0 as a measure of the propagation velocity direction with asterisks indicating significance (95% bootstrap CI, $p < 0.05$).

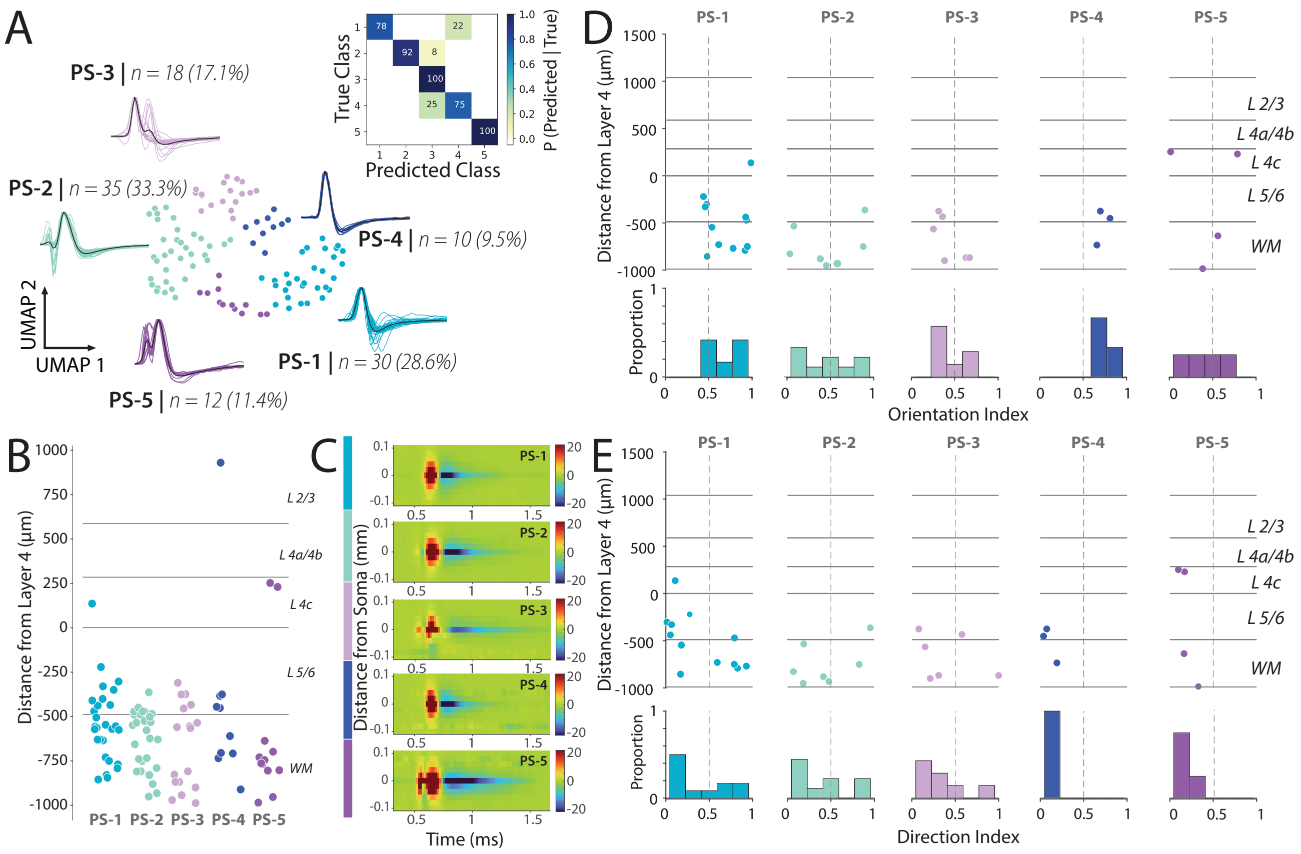

Fig. S6. Positive waveform clusters are largely in white matter and show a diversity of visual responses (**A**) Scatter plot of WaveMAP clustering on 104 positive spiking waveforms (N_neighbors = 20; MIN_DIST = 0.2; RESOLUTION = 1.0) *Inset*, Confusion Matrix showing gradient boosted decision tree classifier with five-fold cross-validation. The main diagonal shows accuracy of waveform classification for each cluster, and off diagonals show misclassification percentages. (**B**) Laminar distribution of 5 positive spiking (PS) clusters across average laminar boundaries. Points are randomly jittered on the x-axis. (**C**) Average multichannel extracellular waveforms per cluster. Cluster color is shown on the left, and the colorbar shows the scale standardized for all clusters to map linearly between -20 and 20 μv. (**D**) Of the 105 positive spiking units, 32 were responsive to visual stimuli. *Top*, Scatter plot of orientation index and scaled laminar depth separated by cluster for positive spiking units. *Bottom*, Panel below the scatter plots shows histograms of the orientation index. The vertical dashed center lines show the boundary between not selective (left of center) and selective (right of center). While the numbers are too small to strong statistical conclusions, clusters PS-1 and PS-4 have strong orientation selectivity but low direction selectivity similar to TP-1 type B neurons (Fig. S5B). (**E**) *Top*, Scatter plot of direction index and scaled laminar depth separated by cluster for positive spiking units. *Bottom*, Panel below the scatter plots shows histograms of the direction index. The vertical dashed center lines show the boundary between not selective (left of center) and selective (right of center). In general, clusters PS1-5 show modest direction selectivity.

Table S1. Distribution of Neuronal Clusters Across Cortical Layers

Layer	Narrow-Spiking (NS)										Tri-phasic (TP)		Broad-Spiking (BS)									
	NS-1		NS-2		NS-3		NS-4		NS Total				BS-1		BS-2		BS-3		BS-4		BS Total	
	n	%	n	%	n	%	n	%	n	%	n	%	n	%	n	%	n	%	n	%	n	%
2/3	6	15.4	4	6.3	5	9.6	5	11.4	20	10.1	3	5.1	29	24.6	14	21.5	28	29.2	12	17.4	83	23.9
4a/4b	19	48.7	28	43.8	25	48.1	23	52.3	95	47.7	12	20.3	24	20.3	10	15.4	13	13.5	8	11.6	55	15.8
4c	13	33.3	29	45.3	10	19.2	7	15.9	59	29.6	19	32.2	52	44.1	36	55.4	40	41.7	9	13.0	137	39.4
5	0	0.0	2	3.1	6	11.5	8	18.2	16	8.0	7	11.9	8	6.8	4	6.2	12	12.5	15	21.7	39	11.2
6	1	2.7	1	1.5	6	11.5	1	2.3	9	4.5	8	13.6	5	4.2	1	1.5	3	3.1	20	29.0	29	8.3
WM	0	0.0	0	0.0	0	0.0	0	0.0	0	0.0	10	16.9	0	0.0	0	0.0	0	0.0	5	7.2	5	1.4
Total	39		64		52		44		199		59		118		65		96		69		348	

References

1. EB Trepka, S Zhu, R Xia, X Chen, T Moore, Functional interactions among neurons within single columns of macaque v1. *Elife* **11**, e79322 (2022).
2. C Nicholson, JA Freeman, Theory of current source-density analysis and determination of conductivity tensor for anuran cerebellum. *J. Neurophysiol.* **38**, 356–368 (1975).
3. LA Zhang, P Li, EM Callaway, High-resolution laminar identification in macaque primary visual cortex using neuropixels probes. *eLife* **13**, RP97290 (2024).
4. S Zhu, R Xia, X Chen, T Moore, Heterogeneity of neuronal populations within columns of primate V1 revealed by high-density recordings. *bioRxiv* p. 424048 (2020) Preprint.
5. M Pachitariu, NA Steinmetz, SN Kadir, M Carandini, KD Harris, Fast and accurate spike sorting of high-channel count probes with kilosort. *Adv. Neural Inf. Process. Syst.* **29** (2016).
6. EM Trautmann, et al., Large-scale high-density brain-wide neural recording in nonhuman primates. *Nat. neuroscience* **28**, 1562–1575 (2025).
7. K Lee, N Carr, A Perliss, C Chandrasekaran, Wavemap for identifying putative cell types from in vivo electrophysiology. *STAR protocols* **4**, 102320 (2023).
8. EK Lee, et al., Non-linear dimensionality reduction on extracellular waveforms reveals cell type diversity in premotor cortex. *Elife* **10**, e67490 (2021).
9. V Poulin, F Théberge, Ensemble clustering for graphs: comparisons and applications. *Appl. Netw. Sci.* **4**, 51 (2019).
10. M Sundqvist, J Chiquet, G Rigaill, Adjusting the adjusted rand index: A multinomial story. *Comput. Stat.* **38**, 327–347 (2022).
11. NV Swindale, Orientation tuning curves: empirical description and estimation of parameters. *Biol. cybernetics* **78**, 45–56 (1998).
12. DH Hubel, TN Wiesel, Receptive fields of single neurones in the cat's striate cortex. *The J. physiology* **148**, 574 (1959).
13. JA Movshon, ID Thompson, DJ Tolhurst, Receptive field organization of complex cells in the cat's striate cortex. *The J. Physiol.* **283**, 79–99 (1978).
14. X Jia, et al., High-density extracellular probes reveal dendritic backpropagation and facilitate neuron classification. *J. Neurophysiol.* **121**, 1831–1847 (2019).
15. Y Wei, et al., Associations between in vitro, in vivo and in silico cell classes in mouse primary visual cortex. *Nat. Commun.* **14**, 2344 (2023).
16. Z Ye, et al., Ultra-high-density neuropixels probes improve detection and identification in neuronal recordings. *Neuron* **113**, 3966–3982.e12 (2025).
17. JM Alonso, LM Martinez, Functional connectivity between simple cells and complex cells in cat striate cortex. *Nat. Neurosci. 1998 1:5* **1**, 395–403 (1998).
18. A Nandi, et al., Single-neuron models linking electrophysiology, morphology, and transcriptomics across cortical cell types. *Cell Reports* **40**, 111176 (2022).
19. L Estebanez, D Hoffmann, BC Voigt, JFA Poulet, Parvalbumin-Expressing GABAergic Neurons in Primary Motor Cortex Signal Reaching. *Cell Reports* **20**, 308–318 (2017).
20. M Medalla, et al., Multimodal characterization of optogenetic approaches for assessing inhibitory neuron function in macaque monkeys in *Society for Neuroscience Annual Meeting.* (MCP Hall A), (2024) Session PSTR430 - Optogenetic Tools, Presentation PSTR430.09/Y8.
21. JK Mich, et al., Functional enhancer elements drive subclass-selective expression from mouse to primate neocortex. *Cell Reports* **34**, 108754 (2021).
22. S Someck, et al., Positive and biphasic extracellular waveforms correspond to return currents and axonal spikes. *Commun. Biol.* **6**, 950 (2023).
23. J Sibille, et al., High-density electrode recordings reveal strong and specific connections between retinal ganglion cells and midbrain neurons. *Nat. Commun. 2022 13:1* **13**, 1–18 (2022).
24. SH Sun, et al., Analysis of extracellular spike waveforms and associated receptive fields of neurons in cat primary visual cortex. *The J. Physiol.* **599**, 2211–2238 (2021).
